# Mild aortic regurgitation as an independent predictor of major adverse cardiovascular events in non-ST-elevation acute coronary syndrome: insights from a multicentre propensity score-matched cohort study

**DOI:** 10.7189/jogh.16.04404

**Published:** 2026-11-13

**Authors:** Peng Zhao, Wenqiang Fang, Hui Li, Bowen Zhou, Tongjian Zhu, Jiajun Zhu, Long Tang, Shili Wu, Bing Wu, Jun Wang, Jinjun Liu

**Affiliations:** 1 Department of Cardiology, The First Affiliated Hospital of Bengbu Medical University, Bengbu, Anhui, China; 2 Joint Research Center for Regional Diseases of IHM, Bengbu Medical University, Bengbu, Anhui, China; 3 Joint Research Center for Regional Diseases of IHM, The First Affiliated Hospital of Bengbu Medical University, Bengbu, Anhui, China; 4 Department of Cardiology, Suzhou First People’s Hospital, Suzhou, Anhui, China; 5 Department of Cardiology, Xiangyang Central Hospital, Xiangyang, Hubei, China; 6 Cardiac Care Unit, First Affiliated Hospital of Xinjiang Medical University, Urumchi, China; 7 Department of Cardiology, The Affiliated Xuancheng Hospital of Wannan Medical University, Xuancheng, Anhui, China; 8 Department of Cardiology, The People’s Hospital of Bozhou, Bozhou, Anhui, China; 9 Institute of Clinical Medicine and Department of Cardiology, Renmin Hospital, Hubei University of Medicine, Shiyan, Hubei, China; 10 Hubei Clinical Medical Research Center for Atherosclerotic Cardiovascular Diseases, Shiyan, Hubei, China; 11 Shiyan Key Laboratory for Atherosclerotic Disease Research, Renmin Hospital Hubei University of Medicine, Shiyan, Hubei, China

**Keywords:** non-ST-elevation acute coronary syndrome, mild aortic regurgitation, major adverse cardiovascular events, inflammation, remnant cholesterol

## Abstract

**Background:**

Non-ST-elevation acute coronary syndrome (NSTE-ACS) remains a leading cause of cardiovascular morbidity and mortality worldwide, making accurate risk stratification beyond conventional clinical parameters a critical need. Mild aortic regurgitation (AR) is frequently detected on routine echocardiography in patients with coronary artery disease, yet its prognostic significance in the high-risk context of NSTE-ACS remains poorly defined.

**Methods:**

This multicentre retrospective cohort study included 1,290 patients with NSTE-ACS and mild AR from six tertiary cardiovascular centres in China. Propensity score matching targeting an approximately 1:3 ratio identified 3,872 matched controls without AR or other valvular heart disease. The primary endpoint was major adverse cardiovascular events (MACEs), comprising cardiac death, heart failure rehospitalisation, and unplanned coronary revascularisation, over a median follow-up of 50 months.

**Results:**

Covariates included in the propensity-score model were adequately balanced after propensity score matching (absolute standardised mean difference <0.10). Mild AR was associated with higher risks of MACEs (HR = 1.47; 95% CI = 1.26–1.72, *P* < 0.001), cardiac death (HR = 1.51; 95% CI = 1.13–2.02, *P* = 0.002), heart failure rehospitalisation (HR = 1.57; 95% CI = 1.12–2.20, *P* = 0.004), and unplanned coronary revascularisation (HR = 1.34; 95% CI = 1.08–1.67, *P* = 0.004). After multivariable adjustment, mild AR remained independently associated with MACEs (HR = 1.329; 95% CI = 1.144–1.545, *P* < 0.001). Mild AR was also associated with higher neutrophil-to-lymphocyte ratio (*β* = 0.56; 95% CI = 0.37–0.74, *P* < 0.001), systemic immune-inflammation index (*β* = 161.73; 95% CI = 119.36–204.10, *P* < 0.001), and remnant cholesterol (*β* = 0.21; 95% CI = 0.16–0.26, *P* < 0.001). Exploratory mediation analyses showed modest statistical indirect associations between mild AR and MACEs (~6.8%) and between mild AR and cardiac death (~10.0%), operating through left atrial diameter.

**Conclusions:**

In patients with NSTE-ACS, mild AR was independently associated with MACEs and modest differences in inflammatory indices and remnant cholesterol. Mild AR may, therefore, be a readily available marker for risk stratification in this high-risk population.

Non-ST-elevation acute coronary syndrome (NSTE-ACS) represents the most prevalent form of acute coronary syndrome and remains a leading cause of cardiovascular morbidity and mortality worldwide [1–4]. Despite advances in pharmacological therapy and percutaneous coronary intervention (PCI), long-term outcomes remain heterogeneous, with a considerable proportion of patients experiencing recurrent ischaemic events, heart failure (HF) rehospitalisation, and cardiac death during long-term follow-up [5]. Current risk stratification tools, including the Global Registry of Acute Coronary Events (GRACE) scores and Thrombolysis in Myocardial Infarction risk scores, primarily integrate conventional clinical, electrocardiographic, and biomarker parameters, yet their discriminatory performance for long-term outcomes beyond the index hospitalisation remains suboptimal [6,7]. This highlights the importance of identifying novel, clinically accessible risk markers to refine prognostic stratification in this high-risk population.

Aortic regurgitation (AR) is among the most frequently encountered valvular abnormalities in clinical cardiology, with the reported prevalence of mild AR reaching up to 13% in the general population and being substantially higher among older individuals and patients with established coronary artery disease (CAD) [8,9]. Traditionally, mild AR has been considered a hemodynamically insignificant valvular abnormality requiring neither pharmacological intervention nor surveillance beyond routine echocardiographic monitoring [10–13]. However, emerging evidence from high-risk cardiovascular cohorts has begun to challenge this view: in patients with acute HF and preserved ejection fraction, concomitant non-severe AR has been independently associated with increased short-term mortality [14,15]. Similarly, even mild paravalvular AR following transcatheter aortic valve replacement has been associated with poorer long-term survival [16,17]. Beyond these specific settings, chronic diastolic volume overload imposed by mild AR may elevate left ventricular end-diastolic pressure (LVEDP) and impair coronary perfusion, these hemodynamic perturbations could be particularly detrimental in the setting of acute myocardial ischaemia [18,19]. Whether mild AR retains independent prognostic significance in patients with NSTE-ACS and through what mechanisms has not been established.

Systemic inflammation and residual lipid burden play central roles in atherosclerotic plaque instability and adverse outcomes following ACS [20–22]. Inflammatory indices derived from routine blood counts, including the neutrophil-to-lymphocyte ratio (NLR) and the systemic immune-inflammation index (SII), have emerged as predictors of adverse cardiovascular events in patients with CAD [23,24]. Remnant cholesterol (RC), calculated as total cholesterol minus low-density lipoprotein cholesterol (LDL-C) − high-density lipoprotein cholesterol (HDL-C), has been recognised as a mediator of residual cardiovascular risk beyond conventional lipid parameters [25,26]. Notably, chronic hemodynamic stress imposed by mild AR may promote endothelial dysfunction, leukocyte adhesion, and inflammatory cytokine release [27,28] pathways that could amplify both systemic inflammatory activity and atherogenic lipid dysregulation. Whether mild AR in patients with NSTE-ACS is independently associated with heightened inflammatory and metabolic risk profiles, and whether such profiles contribute to the mechanisms linking mild AR to adverse outcomes, has not been formally investigated.

Accordingly, we hypothesised that mild AR may be associated with adverse cardiovascular outcomes and modest differences in inflammatory and lipid-related profiles in patients with NSTE-ACS. To test this hypothesis, we conducted a multicentre propensity score-matched cohort study to determine the independent association of mild AR with MACEs in patients with NSTE-ACS, characterise the relationship between mild AR and systemic inflammatory indices and remnant cholesterol, and explore the potential mediating role of cardiac structural remodelling in these associations.

## METHODS

In this multicentre retrospective cohort study, we included patients with NSTE-ACS admitted to six tertiary cardiovascular centres in eastern, western, and central China between January 2018 and December 2020. Patients with concomitant mild AR constituted the exposed group, whereas those without echocardiographically diagnosed AR or other valvular heart disease (VHD) served as the comparator group.

We considered patients who met all of the following criteria: age ≥18 years; confirmed diagnosis of NSTE-ACS, encompassing unstable angina and non-ST-segment elevation myocardial infarction (MI), according to the 2020 European Society of Cardiology (ESC) Guidelines [29]; and successful coronary angiography (CAG) performed during the index hospitalisation. We excluded patients who had inconclusive or technically uninterpretable CAG imaging; severe renal insufficiency (estimated glomerular filtration rate <30 mL/min/1.73 m^2^) or hepatic insufficiency (Child-Pugh class B or C); concomitant moderate-to-severe AR or any other significant VHD; active malignancy or systemic autoimmune disease at the time of enrolment; or incomplete data for any variable included in the propensity score model. Patients with mild AR were the exposed group, whereas while without echocardiographically diagnosed AR or other VHD were the comparator group.

We diagnosed NSTE-ACS according to current international guidelines [29] and evaluated the severity of AR by transthoracic echocardiography using a comprehensive multiparameter approach, consistent with the ESC Guidelines for the Management of VHD [30]. Here, we defined mild AR according to established echocardiographic criteria [18], including vena contracta width, pressure half-time, and colour Doppler jet area, with AR severity graded at each participating centre using a standardised multiparametric transthoracic echocardiographic protocol. All participating centres implemented standardised diagnostic and treatment protocols for ACS throughout the study period.

### Data collection and biomarker definitions

We systematically reviewed the electronic medical records of all enrolled patients to verify diagnoses and to retrieve demographic characteristics, cardiovascular risk factors, comorbidities, medication history, laboratory parameters, echocardiographic data, and CAG findings. To minimise the influence of procedural stress on circulating biomarker levels, we collected venous blood samples within 24 hours of admission and prior to CAG. All samples were processed in accordance with standardised laboratory protocols at each participating centre.

We calculated systemic inflammatory indices from routine peripheral blood cell counts [23,24]. We defined NLR as the neutrophil count divided by the lymphocyte count, with the platelet-to-lymphocyte ratio calculated as the platelet count divided by the lymphocyte count, and the monocyte-to-lymphocyte ratio calculated as the monocyte count divided by the lymphocyte count. We derived the SII using the ‘SII = (platelet count × neutrophil count)/lymphocyte count’ and the RC using the formula ‘RC = total cholesterol − LDL-C − HDL-C’ [25,26].

### Follow-up and outcomes

We followed up all patients for up to 50 months after the index hospitalisation, with the data on follow-ups obtained from hospital electronic medical records, outpatient visit records, and structured telephone interviews conducted at predetermined intervals by trained research coordinators.

The primary endpoint was the occurrence of major adverse cardiovascular events (MACEs) during the follow-up period. Here, MACEs were defined as a composite of: cardiac death, defined as death attributable to a cardiovascular cause, including sudden cardiac death, death due to acute MI, NF, or other cardiac causes; HF rehospitalisation, defined as an unplanned hospital admission primarily due to worsening HF requiring intravenous therapy; and unplanned coronary revascularisation, defined as any unscheduled repeat PCI or coronary artery bypass grafting following the index hospitalisation. All candidate endpoint events were independently reviewed and classified according to prespecified endpoint definitions to determine whether they met the criteria for cardiac death, HF rehospitalisation, or unplanned coronary revascularisation. The reviewers were blinded to patients’ baseline characteristics and study group assignment.

### Missing data handling

We used a complete-case approach where we excluded all patients with missing data for any required variable from our analyses, meaning we performed no imputation. We did not formally assess variable-specific pattern and mechanism of missingness.

### PSM

We performed PSM using a nearest-neighbour matching algorithm without replacement, with a calliper width of 0.02 standard deviations (SDs) of the logit of the propensity score, targeting an approximately 1:3 exposed-to-control matching ratio. We estimated propensity scores using a multivariable logistic regression model that incorporated the following covariates: age, sex, body mass index (BMI), hypertension, diabetes mellitus, smoking history, alcohol consumption history, family history of CAD, previous MI, ACS subtype, baseline medications (aspirin, clopidogrel, ticagrelor, statins, beta-blockers, angiotensin-converting enzyme inhibitors, angiotensin receptor blockers, calcium channel blockers, angiotensin receptor–neprilysin inhibitors). We assessed covariate balance after matching using absolute standardised mean differences (SMDs), rather than *P*-values. For continuous variables, the SMD was calculated as the absolute difference in group means divided by the square root of the average of the two group variances; for binary variables, the difference in proportions was standardised by the square root of the average binomial variance. We considered an absolute SMD <0.10 as indicative of adequate balance.

### Statistical analysis

We summarised continuous variables as means (x̄) and SDs for normally distributed data and as medians (Mdn) and interquartile ranges (IQRs) for non-normally distributed data, as determined by the Shapiro–Wilk test, with between-group comparisons performed using independent samples *t*-tests or Mann–Whitney U tests, respectively. We otherwise presented categorical variables as frequencies and percentages and conducted between-group comparisons using chi-squared tests or Fisher’s exact tests, as appropriate.

We constructed Kaplan–Meier survival curves to estimate the cumulative incidence of MACEs and its individual components, with between-group differences evaluated using the log-rank test. We first performed univariable Cox proportional hazards regression analyses to identify candidate predictors, followed by multivariable Cox regression with adjustment for clinically relevant covariates to estimate adjusted hazard ratios (HRs) and their corresponding 95% confidence intervals (CIs).

We then performed subgroup analyses to assess the consistency of the association between mild AR and MACEs across subgroups. *P*-values for interaction were calculated using likelihood ratio tests comparing models with and without an interaction term. To evaluate independent associations between mild AR and systemic inflammatory indices (NLR and SII) and RC, we constructed separate multivariable linear regression models with NLR, SII, and RC as dependent variables, respectively, with Pearson or Spearman’s correlation coefficients calculated to further characterise these associations, as appropriate. Lastly, we performed mediation analyses to investigate possible indirect mechanisms linking mild AR to MACEs. The mediation analyses quantified the magnitude of indirect effects and provided corresponding 95% CIs.

We performed all statistical analyses using *R*, version 4.2.2 (R Foundation for Statistical Computing, Vienna, Austria), with the ‘MatchIt’, ‘survival’, ‘mediation’, and ‘ggplot2’ packages used for PSM, survival analysis, mediation analysis, and data visualisation, respectively. Statistical significance was defined as a two-tailed *P*-value <0.05.

## RESULTS

Among 5,820 otherwise eligible patients, 417 (7.2%) were excluded because of incomplete data, leaving 5,403 eligible participants. After PSM targeting an approximately 1:3 ratio, the matched cohort comprised 1,290 patients with NSTE-ACS and mild AR and 3,872 matched controls with NSTE-ACS but without VHD (**Figure 1**). Following PSM, all covariates included in the propensity-score model achieved adequate balance (absolute SMDs <0.10) (**Table 1**). *P*-values are retained only for descriptive comparisons and were not used to judge covariate balance. 

**Figure 1:**
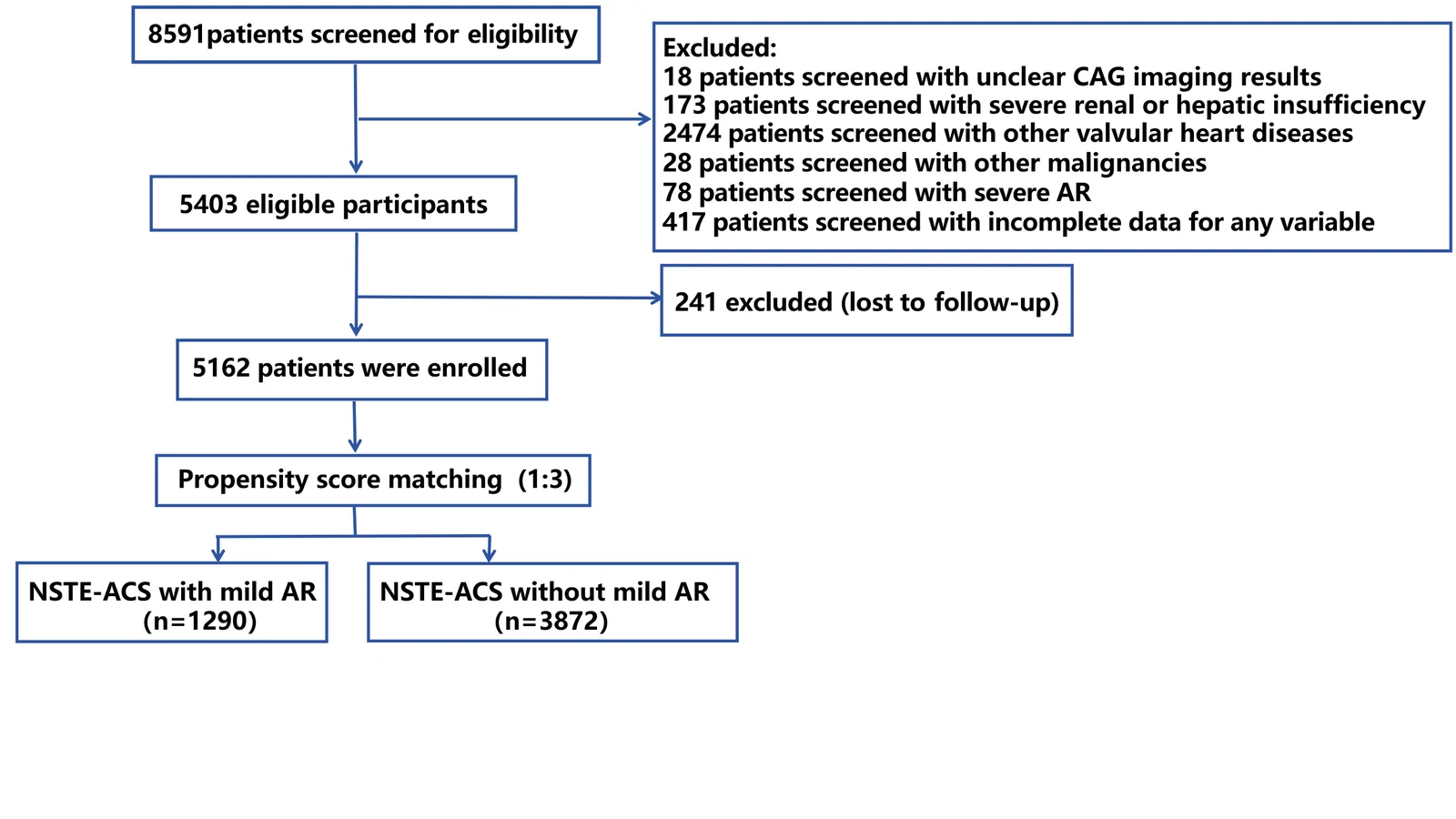
Flowchart of the study.

**Table 1:** Baseline characteristics and clinical outcomes

	NSTE-ACS without AR or other VHD (n = 3872)	NSTE-ACS with mild AR (n = 1290)	*P*-value	SMD
**Male sex**	2428 (62.7)	784 (60.8)	0.215	0.040
**Age in years, x̄ (SD)**	63.76 (11.62)	63.80 (10.87)	0.895	0.004
**Hypertension**	2574 (66.5)	838 (65.0)	0.319	0.032
**Diabetes mellitus**	1173 (30.3)	409 (31.7)	0.341	0.031
**Smoking history**	1265 (32.7)	414 (32.1)	0.701	0.012
**Alcohol consumption history**	963 (24.9)	317 (24.6)	0.830	0.007
**Family history of CAD**	149 (3.8)	54 (4.2)	0.589	0.017
**Previous MI**	171 (4.4)	61 (4.7)	0.639	0.015
**Subtypes of acute coronary syndromes**			0.220	0.039
Unstable angina	3449 (89.1)	1133 (87.8)		
Non-ST-segment elevation MI	423 (10.9)	157 (12.2)		
**BMI in kg/m^2^, x̄ (SD)**	24.91 (3.30)	24.87 (3.09)	0.688	0.013
**Use of aspirin**	3118 (80.5)	1026 (79.5)	0.438	0.025
**Use of clopidogrel**	645 (16.7)	232 (18.0)	0.272	0.035
**Use of ticagrelor**	47 (1.2)	20 (1.6)	0.355	0.029
**Use of statins**	3007 (77.7)	980 (76.0)	0.210	0.040
**Use of beta blockers**	1873 (48.4)	596 (46.2)	0.176	0.044
**Use of ACEI**	416 (10.7)	145 (11.2)	0.620	0.016
**Use of ARB**	830 (21.4)	264 (20.5)	0.460	0.024
**Use of CCB**	969 (25.0)	356 (27.6)	0.067	0.058
**Use of ARNI**	477 (12.3)	164 (12.7)	0.710	0.012
**SBP in mmHg, x̄ (SD)**	140.64 (23.11)	140.43 (22.86)	0.777	0.009
**DBP in mmHg, x̄ (SD)**	84.10 (14.71)	84.12 (14.17)	0.963	0.002
**White blood count × 10^9^/L, x̄ (SD)**	6.85 (2.35)	6.82 (2.42)	0.684	0.013
**Neutrophil count × 10^9^/L, Mdn (IQR)**	4.00 (3.15–5.37)	4.09 (3.22–5.52)	0.021	0.111
**Lymphocyte count × 10^9^/L, Mdn (IQR)**	1.61 (1.23–2.07)	1.56 (1.18–2.02)	0.010	0.095
**Monocyte count × 10^9^/L, Mdn (IQR)**	0.40 (0.29–0.54)	0.42 (0.28–0.56)	0.155	0.058
**Platelet count × 10^9^/L, x̄ (SD)**	206.08 (62.95)	212.27 (64.87)	0.002	0.097
**NLR, Mdn (IQR)**	2.44 (1.77–3.76)	2.63 (1.83–4.05)	<0.001	0.173
**SII, Mdn (IQR)**	488.68 (328.69–779.34)	526.16 (356.32–850.59)	<0.001	0.207
**PLR, Mdn (IQR)**	122.56 (92.90–166.11)	132.14 (96.53–176.54)	<0.001	0.194
**MLR, Mdn (IQR)**	0.24 (0.17–0.34)	0.26 (0.18–0.37)	0.003	0.135
**Creatinine in *μ*mol/L, x̄ (SD)**	74.69 (25.84)	75.35 (26.95)	0.429	0.025
**Uric acid in *μ*mol/L, x̄ (SD)**	341.34 (106.71)	345.32 (118.90)	0.286	0.035
**Fasting blood glucose in mmol/L, x̄ (SD)**	6.86 (3.24)	6.78 (3.03)	0.457	0.023
**Heart rate as times per minute, x̄ (SD)**	75.91 (13.43)	74.92 (12.52)	0.015	0.077
**Total cholesterol in mmol/L, x̄ (SD)**	4.66 (1.00)	4.68 (1.01)	0.550	0.019
**TG in mmol/L, Mdn (IQR)**	1.59 (1.06–2.17)	1.47 (1.02–1.90)	<0.001	0.169
**HDL-C in mmol/L, x̄ (SD)**	1.10 (0.30)	1.07 (0.32)	0.001	0.110
**LDL-C in mmol/L, x̄ (SD)**	2.48 (0.93)	2.33 (0.98)	<0.001	0.160
**RC in mmol/L, Mdn (IQR)**	0.89 (0.52–1.25)	0.92 (0.63–1.66)	<0.001	0.238
**Fibrinogen in g/L, Mdn (IQR)**	2.98 (2.39–3.67)	3.01 (2.49–3.78)	0.048	0.022
**Left ventricular ejection fraction in %, x̄ (SD)**	56.70 (6.52)	51.54 (11.20)	<0.001	0.564
**LVEDD in mm, x̄ (SD)**	47.78 (4.84)	48.86 (6.14)	<0.001	0.196
**Left atrial diameter on mm, x̄ (SD)**	37.51 (4.81)	38.72 (4.49)	<0.001	0.260
**Percutaneous coronary intervention**	938 (24.2)	316 (24.5)	0.844	0.006
**Number of implanted stents**	0.00(0.00–1.00)	0.00 (0.00–1.00)	<0.001	0.139
**The number of coronary arteries with ≥50% stenosis**	1.00(1.00–1.00)	1.00 (1.00–2.00)	<0.001	0.281
MACEs	594 (15.3)	275 (21.3)	<0.001	
Cardiac death	162 (4.2)	80 (6.2)	0.003	
Revascularisation	311 (8.0)	134 (10.4)	0.009	
Heart failure rehospitalisation	121 (3.1)	61 (4.7)	0.007	

ACEI – angiotensin-converting enzyme inhibitor, AR – aortic regurgitation, ARB – angiotensin receptor blocker, ARNI – angiotensin receptor neprilysin inhibitor, BMI – body mass index, CAD – coronary artery disease, CCB – calcium channel blocker, DBP – diastolic blood pressure, HDL-C – high-density lipoprotein cholesterol, IQR – interquartile range, LDL-C – low-density lipoprotein cholesterol, Mdn – median, MI – myocardial infarction, MLR – monocyte-to-lymphocyte ratio, NLR – neutrophil-to-lymphocyte ratio, NSTE-ACS – non-ST-elevation acute coronary syndrome, PLR – platelet-to-lymphocyte ratio, RC – remnant cholesterol, SBP – systolic blood pressure, SD – standard deviation, SII – systemic immune-inflammation index, SMD – standardised mean difference, TG – triglyceride, VHD – valvular heart disease, x̄ – mean.

*Presented as n (%) unless specified otherwise. Absolute SMDs are reported for baseline characteristics only. For continuous variables, SMD = |mean_mild AR − mean_control|/sqrt((SD_mild AR^2^ + SD_control^2^)/2). For binary variables, SMD = |p_mild AR − p_control|/sqrt({p_mild AR(1 − p_mild AR) + p_control(1 − p_control)}/2). An absolute SMD<0.10 was considered indicative of adequate balance. Outcome rows are not balance measures and, therefore, have no SMD.

Notably, compared with matched controls, patients with mild AR had significantly lower left ventricular ejection fraction (LVEF) (x̄ = 51.54%, SD = 11.20 *vs*. x̄ = 56.70%, SD = 6.52; *P *< 0.001), greater left ventricular end-diastolic dimension (LVEDD) (x̄ = 48.86 mm, SD = 6.14 *vs*. x̄ = 47.78 mm, SD = 4.84; *P* < 0.001), and larger left atrial diameter (LAD) (x̄ = 38.72 mm, SD = 4.49 *vs*. x̄ = 37.51 mm, SD = 4.81; *P* < 0.001). Inflammatory indices were also significantly elevated in the mild AR group, including NLR (Mdn = 2.63, IQR = 1.83–4.05 *vs*. Mdn = 2.44, IQR = 1.77–3.76; *P* < 0.001), SII (Mdn = 526.16, IQR = 356.32–850.59 *vs*. Mdn = 488.68, IQR = 328.69–779.34; *P* < 0.001), platelet-to-lymphocyte ratio (Mdn = 132.14, IQR = 96.53–176.54 *vs*. Mdn = 122.56, IQR = 92.90–166.11; *P* < 0.0019) and monocyte-to-lymphocyte ratio (Mdn = 0.26, IQR = 0.18–0.37 *vs* Mdn = 0.24, IQR = 0.17–0.34; *P* = 0.003). The unadjusted incidence of individual MACEs components was significantly higher in the mild AR group compared with the control group: cardiac death (6.2% *vs*. 4.2%; *P* = 0.003), HF rehospitalisation (4.7% *vs*. 3.1%; *P* = 0.007), and unplanned coronary revascularisation (10.4% *vs*. 8.0%; *P* = 0.009).

### Independent association of Mild AR with MACEs

During a median follow-up of 50 months, patients with mild AR demonstrated a significantly higher cumulative incidence of all prespecified endpoints compared with matched controls (**Figure 2**).

**Figure 2:**
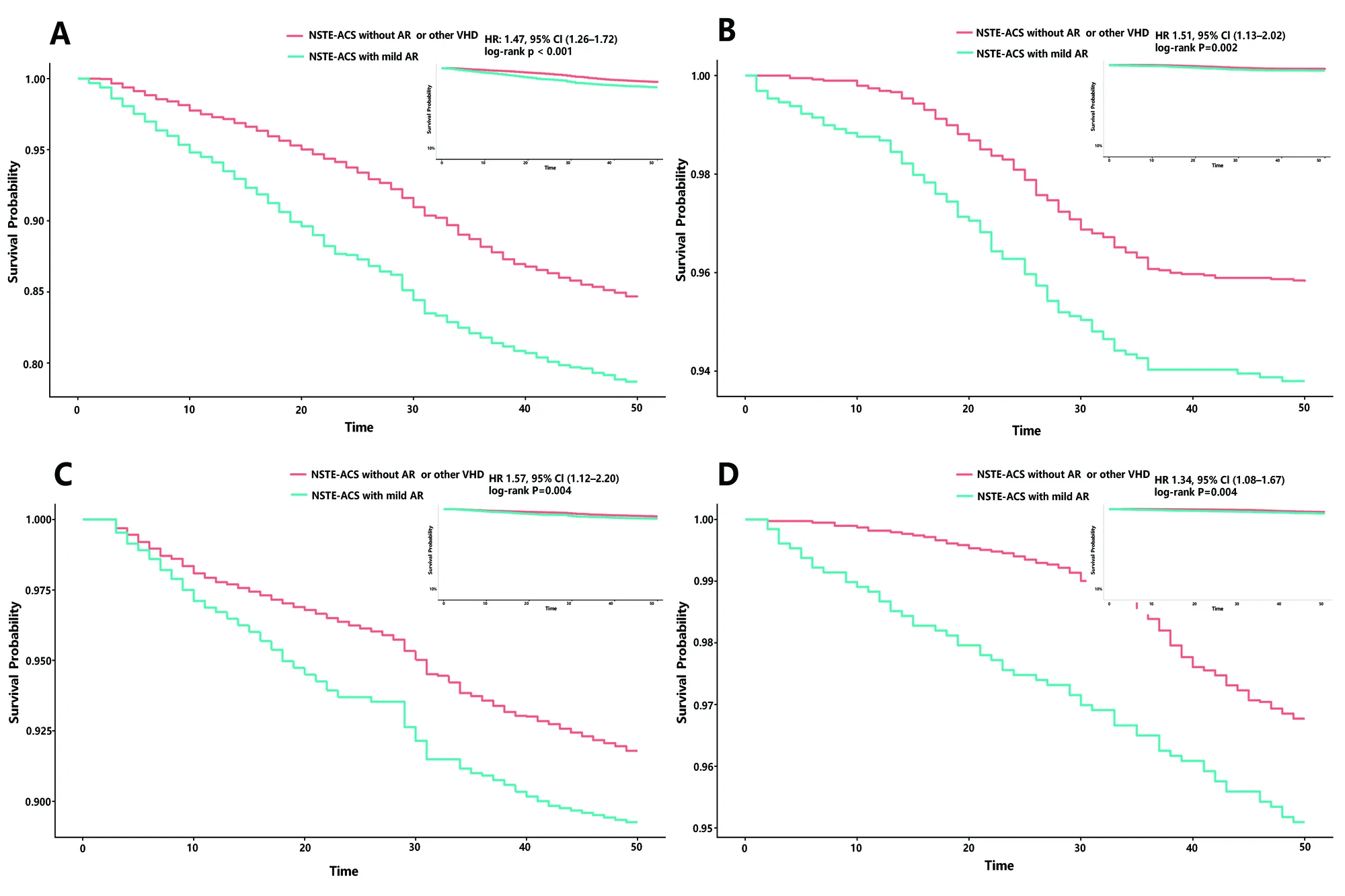
Kaplan-Meier curves were plotted respectively for the occurrence of MACEs (**Panel A**), cardiac death (**Panel B**), HF rehospitalisation (**Panel C**), and unplanned coronary revascularisation (**Panel D**) in NSTE-ACS patients based on whether they had mild aortic valve regurgitation. AR – aortic regurgitation, CI – confidence interval, HR – hazard ratio, MACE – major adverse cardiovascular events, NSTE-ACS – non-ST-elevation acute coronary syndrome, VHD – valvular heart disease.

In unadjusted analyses of the propensity score-matched cohort, mild AR (HR = 1.47; 95% CI = 1.26–1.72, *P* < 0.001) was associated with a higher risk of MACEs compared with controls, with divergence of the event-free survival curves becoming apparent in the early follow-up period and persisting throughout the observation window. Consistent associations were observed across all individual MACEs components: cardiac death (HR = 1.51; 95% CI = 1.13–2.02, *P* = 0.002), HF rehospitalisation (HR = 1.57; 95% CI = 1.12–2.20, *P* = 0.004), and unplanned coronary revascularisation (HR = 1.34; 95% CI = 1.08–1.67, *P* = 0.004). Neither group reached the median event-free survival time during the follow-up period, reflecting the relatively low absolute cumulative event rate in this cohort.

In the univariate Cox regression analyses (Table S1 in the **Online Supplementary Document**), mild AR was significantly associated with an increased risk of MACEs (HR = 1.472; 95% CI = 1.276–1.698, *P* < 0.001) and each of its constituent endpoints in univariable analyses. Among the additional clinical and angiographic variables examined, diabetes mellitus, smoking history, BMI, RC, LAD, creatinine, number of implanted stents, and multivessel CAD (defined as ≥50% stenosis in two or three coronary arteries) were each significantly associated with adverse outcomes on univariable analysis and were, therefore, carried forward as covariates in the multivariable models.

In the multivariable Cox proportional hazards regression analyses (**Figure 3**), mild AR (HR = 1.329; 95% CI = 1.144–1.545, *P* < 0.001), diabetes mellitus (HR = 1.299; 95% CI = 1.130–1.494, *P* < 0.001), smoking history (HR = 1.190; 95% CI = 1.035–1.368, *P* = 0.014), BMI (HR = 1.024; 95% CI = 1.003–1.045, *P* = 0.021), LAD (HR = 1.021; 95% CI = 1.006–1.035, *P* = 0.005), number of implanted stents (HR = 1.143; 95% CI = 1.056–1.236, *P* = 0.001), and the presence of two-vessel (HR = 1.285; 95% CI = 1.032–1.599, *P* = 0.025) or three-vessel CAD (HR = 1.255; 95% CI = 1.045–1.506, *P* = 0.015) were independently associated with an increased risk of MACEs. Two-vessel CAD (HR = 2.269; 95% CI = 1.615–3.188, *P* < 0.001), LAD (HR = 1.038; 95% CI = 1.010–1.067, *P* = 0.007), RC (HR = 1.176; 95% CI = 1.023–1.352, *P* = 0.023), smoking history (HR = 1.362; 95% CI = 1.051–1.765, *P* = 0.019), diabetes mellitus (HR = 1.339; 95% CI = 1.031–1.739, *P* = 0.028), BMI (HR = 1.042; 95% CI = 1.004–1.082, *P* = 0.031), and mild AR (HR = 1.331; 95% CI = 1.002–1.768, *P* = 0.049) were independently associated with an increased risk of cardiac death. Diabetes mellitus (HR = 1.228; 95% CI = 1.011–1.493, *P* = 0.039) and mild AR (HR = 1.250; 95% CI = 1.011–1.546, *P* = 0.039) emerged as independent predictors for HF rehospitalisation. For unplanned coronary revascularisation, mild AR (HR = 1.502; 95% CI = 1.089–2.073, *P* = 0.013), two-vessel CAD (HR = 1.715; 95% CI = 1.122–2.620, *P* = 0.013), number of implanted stents (HR = 1.355, 95% CI = 1.172–1.566, *P* < 0.001), smoking history (HR = 1.471; 95% CI = 1.094–1.979, *P* = 0.011), diabetes mellitus (HR = 1.434; 95% CI = 1.062–1.935, *P* = 0.019), and BMI (HR = 1.045; 95% CI = 1.001–1.091, *P* = 0.045) were independently associated with an increased risk of unplanned coronary revascularisation.

**Figure 3:**
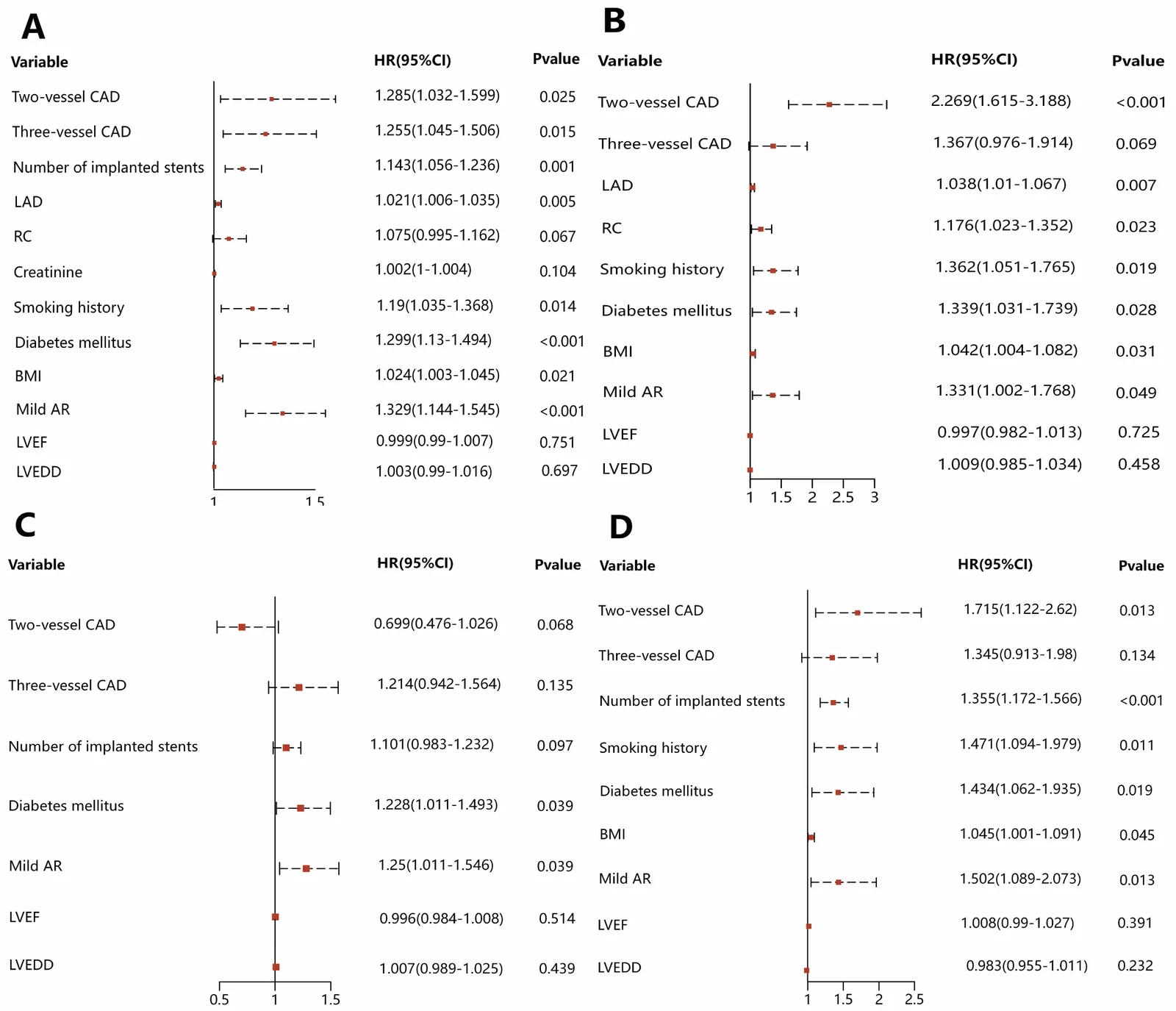
Multivariate Cox regression analysis of MACEs (**Panel A**), cardiac death (**Panel B**), HF rehospitalisation (**Panel C**), and unplanned coronary revascularisation (**Panel D**) in NSTE-ACS patients with mild AR. AR – aortic regurgitation, BMI – body mass index, CAD – coronary artery disease, LAD – left atrial diameter, LVEDD – left ventricular end-diastolic dimension, LVEF – left ventricular ejection fraction, RC – remnant cholesterol.

### Subgroup analysis

The association between mild AR and MACEs was consistent across the majority of predefined subgroups, with no statistically significant interactions detected, supporting the robustness of the primary findings (Figure S1 in the **Online Supplementary Document**). The HR for MACEs associated with mild AR ranged from 1.24 to 1.64 across subgroups defined by age (<65 *vs*. ≥65 years), sex (male *vs*. female), presence of diabetes mellitus, hypertension, smoking history, alcohol consumption history, family history of CAD, previous MI, PCI and ACS subtype (unstable angina *vs*. non-ST-segment elevation MI). No significant interactions were observed across the examined subgroups, with all *P*-values for interaction >0.05, supporting the consistency of the associations between mild AR and multiple adverse cardiovascular outcomes, including MACEs, cardiac death, HF rehospitalisation, and unplanned coronary revascularisation.

### Associations of mild AR with inflammatory indices and remnant cholesterol

After adjustment, mild AR was independently and significantly associated with higher NLR levels (unstandardised regression coefficient (*β*) = 0.56; 95% CI = 0.37–0.74, *P* < 0.001). Among co-predictors, ACS subtype (*β* = 1.61; 95% CI = 1.35–1.86, *P* = 0.026), diabetes mellitus (*β* = 0.27; 95% CI = 0.10–0.44, *P* = 0.002), and heart rate (*β* = 0.01; 95% CI = 0.01–0.02, *P* < 0.001) were positively associated with NLR, while BMI (*β* = −0.03; 95% CI = −0.05–0.00, *P* = 0.026) and DBP (*β* = −0.01; 95% CI = −0.01–0.00, *P* = 0.035) showed inverse associations (Figure S2 in the **Online Supplementary Document**). Sex, age, hypertension, smoking history, alcohol consumption history, family history of CAD, previous MI, previous PCI, and SBP were not independently associated with NLR. In separate multivariable linear regression analyses, mild AR was also independently associated with higher SII levels (*β* = 161.73; 95% CI = 119.36–204.10, *P* < 0.001) and elevated RC (*β* = 0.21; 95% CI = 0.16–0.26, *P* < 0.001) after multivariable adjustment. Correlation analyses showed statistically significant, but weak positive associations of mild AR with NLR (correlation coefficient (r) = 0.083, *P* < 0.001), SII (r = 0.103, *P* < 0.001), and remnant cholesterol (r = 0.109, *P* < 0.001). These coefficients corresponded to shared variances of approximately 0.7%, 1.1%, and 1.2%, respectively, indicating limited effect sizes, despite statistical significance (Figure S3 in the **Online Supplementary Document**).

### Mediation analysis

In exploratory mediation analyses (Table S2 in the **Online Supplementary Document**), LAD showed a statistically significant indirect association between mild AR and both MACEs and cardiac death. The indirect effect of mild AR on MACEs operating through LAD accounted for approximately 6.8% of the total effect, while the mediated proportion for cardiac death was approximately 10.0%; both were statistically significant on bootstrap-based inference (Figure S4 in the **Online Supplementary Document**).

For HF rehospitalisation and unplanned coronary revascularisation, although mild AR was significantly associated with all six candidate cardiac structural parameters at *P* < 0.001, these associations were non-significant in the mediation framework, with all *P*-values for indirect effect >0.05. Nevertheless, the total effects of mild AR on HF rehospitalisation (*β* = 0.023; 95% CI = 0.005–0.041, *P* = 0.011) and unplanned coronary revascularisation (*β* = 0.016; 95% CI = 0.004–0.027, *P* = 0.008) remained statistically significant, as did the direct effects after adjustment for all candidate mediators–HF rehospitalisation (*β* = 0.020; 95% CI = 0.001–0.039, *P* = 0.038) and unplanned revascularisation (*β* = 0.017; 95% CI = 0.005–0.029, *P* = 0.007), indicating that the predominant effect of mild AR on these endpoints operates through pathways not captured by the current mediation framework.

## DISCUSSION

In this multicentre propensity score–matched cohort study, we investigated the prognostic significance of mild AR in patients with NSTE-ACS and yielded three principal findings. First, patients with NSTE-ACS and concomitant mild AR exhibited significantly higher risks of MACEs and its individual components, including cardiac death, HF rehospitalisation, and unplanned coronary revascularisation. Notably, mild AR remained independently associated with an increased risk of MACEs after multivariable adjustment. Second, mild AR was independently associated with elevated inflammatory indices, including NLR and SII. Third, mild AR was independently associated with elevated RC levels. These findings challenge the traditional perception of mild AR as a hemodynamically inconsequential echocardiographic finding and position it as a clinically meaningful risk marker in patients with NSTE-ACS.

### Mild AR as an independent prognostic marker in NSTE-ACS

Mild AR is among the most prevalent valvular abnormalities encountered in patients with established CAD, yet its prognostic significance in the acute ischaemic context has not been systematically characterised. Clinical practice has traditionally focused on the assessment, management, and timing of surgical intervention for moderate-to-severe AR, while mild AR has generally been regarded as a benign or physiological valve abnormality requiring no specific intervention [10,12,31]. However, emerging evidence indicates that even non-severe valvular abnormalities may have important prognostic implications in high-risk cardiovascular populations [16,32,33].

In patients with acute decompensated HF and preserved ejection fraction, non-severe AR has been independently associated with increased short-term mortality, suggesting that even modest regurgitant volumes may amplify hemodynamic vulnerability when cardiac reserve is impaired [14,15]. Similarly, residual mild paravalvular AR following transcatheter aortic valve replacement has consistently been associated with worse long-term survival [16,17]. Our study extends this paradigm to the NSTE-ACS population, demonstrating for the first time that the adverse prognostic significance of mild AR is not confined to HF or post-procedural settings, but extends to the high-risk context of acute myocardial ischaemia.

The magnitude of the association observed here is clinically noteworthy. After multivariable adjustment for established cardiovascular risk factors, CAD severity, and cardiac structural parameters, mild AR was associated with a 33% higher relative risk of MACEs – an effect size comparable to, and in some cases exceeding, that of established risk markers such as diabetes mellitus and multivessel CAD in this cohort. The association was directionally consistent across the individual MACEs components, suggesting that the observed prognostic relationship may involve multiple underlying pathways rather than a single mechanism. Importantly, because transthoracic echocardiography is already routinely performed during the index hospitalisation for ACS, the identification of concomitant mild AR requires no additional diagnostic testing or cost, making it a readily accessible addition to existing risk stratification frameworks such as the GRACE score. Future studies should formally evaluate whether incorporating echocardiographic AR grading into these tools can improve their discriminatory capacity.

### Hemodynamic mechanisms linking mild AR to adverse outcomes

Several potential mechanisms, which are not mutually exclusive, may underlie the observed association between mild AR and adverse outcomes in patients with NSTE-ACS. Even in its mildest hemodynamically quantifiable form, AR imposes chronic diastolic volume overload on the left ventricle, increasing end-diastolic volume and wall stress [11,34]. Under physiological conditions, the left ventricle can compensate through eccentric hypertrophy and augmented stroke volume. However, in the setting of acute myocardial ischaemia, where contractility is acutely impaired and left ventricular compliance is reduced, even modest additional diastolic loading may push a vulnerable myocardium beyond its adaptive reserve, precipitating or exacerbating hemodynamic decompensation. This mechanism provides a plausible explanation for the higher incidence of both HF rehospitalisation and cardiac death observed in the mild AR group. A second mechanism involves impaired coronary perfusion pressure. Coronary blood flow occurs predominantly during diastole and is determined by the gradient between aortic diastolic pressure and LVEDP. Regurgitant flow across the aortic valve during diastole reduces effective aortic diastolic pressure while simultaneously elevating LVEDP, resulting in a compounded reduction in coronary perfusion pressure [35]. In patients with NSTE-ACS, in whom coronary perfusion is already compromised by plaque instability, subtotal occlusion, and microvascular dysfunction, this superimposed reduction may further aggravate subendocardial ischaemia, promote adverse left ventricular remodelling, and increase the likelihood of unplanned revascularisation. A third consideration relates to the structural substrate underlying mild AR itself. In older patients with CAD, mild AR frequently reflects aortic root dilation, age-related leaflet thickening, or early degenerative valvular changes rather than an isolated functional abnormality [36,37]. These structural alterations indicate a greater overall burden of cardiovascular disease and a more advanced arterial aging process, which may independently predispose affected patients to adverse cardiovascular events. In this context, mild AR may serve as an echocardiographic indicator of subclinical structural vulnerability that is not captured by conventional angiographic or laboratory-based risk markers. Finally, mild AR may exacerbate hemodynamic instability in patients with ischaemic myocardium through increased left ventricular filling pressures and impaired diastolic function, predisposing patients to HF development [33], and thereby contributing to the higher incidence of HF rehospitalisation observed in our study.

### Inflammatory and metabolic pathways

Beyond hemodynamic mechanisms, a particularly notable finding of our analysis is the independent association between mild AR and markers of heightened systemic inflammatory activity and residual lipid burden – an observation that delineates a pro-inflammatory, pro-atherogenic host state as a potential mechanistic link between mild AR and adverse cardiovascular outcomes.

Inflammation plays a central role in the development and progression of atherosclerosis and in the pathogenesis of ACS [38–41]. The NLR and SII are well-validated surrogates of systemic inflammatory activation that have demonstrated robust prognostic value in patients with CAD [41,42]. In multivariable linear regression analysis, mild AR was independently associated with higher NLR and SII levels. However, the correlation between mild AR and SII was weak (r = 0.103), suggesting that the observed association, although statistically significant, was modest in magnitude. Therefore, the clinical relevance of this association should be interpreted cautiously.

Several non-mutually exclusive explanations may underlie the modest associations between mild AR and inflammatory markers. Hemodynamic alterations accompanying valvular disease could potentially contribute to endothelial dysfunction and inflammatory activation [43–45], although shear stress, endothelial function, and related biological pathways were not directly assessed in this study. Alternatively, a greater burden of coronary atherosclerosis, acute myocardial injury, or ischaemia-related cardiac remodelling may be associated with both mild AR and increased inflammatory activity. Because AR severity and inflammatory markers were assessed during the same hospitalisation, temporal directionality cannot be established, and reverse causation or residual confounding cannot be excluded. These findings should, therefore, be regarded as hypothesis-generating, rather than evidence that mild AR directly promotes systemic inflammation.

The independent association between mild AR and elevated RC levels provides an additional mechanistic dimension. Specifically, RC – representing cholesterol contained in triglyceride-rich remnant lipoproteins – has emerged as an important mediator of residual cardiovascular risk beyond LDL-C, promoting endothelial dysfunction, vascular inflammation, and accelerated atherosclerotic plaque progression [46,47]. Mechanistically, RC can readily penetrate the arterial intima, triggering inflammatory signalling cascades and subsequent immune cell activation, and accumulate within the arterial wall to promote inflammatory activation and atherosclerotic progression [48–50]. The co-existence of elevated RC and heightened systemic inflammatory activity in patients with mild AR suggests a mutually reinforcing pro-atherogenic milieu, whereby lipid-driven inflammatory activation and hemodynamic stress may interact synergistically to drive atherosclerotic disease progression and increase coronary plaque vulnerability. Importantly, these associations persisted after adjustment for diabetes mellitus and other established confounders, implying that they are not merely reflective of shared risk factors, but may reflect distinct pathophysiological consequences of the hemodynamic perturbations imposed by mild AR itself.

From a therapeutic standpoint, the identification of this concurrent inflammatory and metabolic dysregulation in patients with mild AR raises a clinically important hypothesis: this subgroup may derive disproportionate benefit from more intensive anti-inflammatory and lipid-lowering strategies-for example, high-intensity statin therapy targeting elevated RC, or adjunctive colchicine therapy targeting residual inflammatory risk-strategies for which there is growing trial-level evidence in the post-ACS setting [51,52]. While the current observational design precludes causal inference regarding treatment effects, this hypothesis is biologically plausible and warrants prospective evaluation.

### Exploratory association involving left atrial diameter

Exploratory mediation analyses identified LAD as a potential statistical mediator of the associations between mild AR and both MACEs and cardiac death. However, because we measured mild AR and LAD concurrently at baseline, we cannot establish the temporal sequence required for causal mediation. In our analysis, LAD emerged as the only statistically significant mediator, partially mediating the association between mild AR and both MACEs (indirect effect proportion ~6.8%) and cardiac death (~10.0%). These findings are mechanistically coherent. Chronic diastolic volume overload from AR leads to progressive LVEDP elevation and impaired diastolic function, which transmits elevated filling pressures to the left atrium, resulting in left atrial remodelling and enlargement over time. Left atrial enlargement is a well-established marker of chronically elevated filling pressures and has been independently associated with atrial fibrillation, HF, and cardiovascular death in multiple large cohort studies [53,54]. These findings are therefore compatible with, but do not demonstrate a potential relationship among mild AR, cardiac structural abnormalities, and subsequent outcomes. Therefore, they should, too, be considered hypothesis-generating. However, the predominantly non-significant indirect effects observed for HF rehospitalisation and unplanned revascularisation, despite significant total and direct effects, indicate that the mechanistic pathways linking mild AR to these specific outcomes extend well beyond left atrial structural remodelling alone. Alternative candidates, including dynamic changes in coronary microvascular function, diastolic pressure–time integral impairment, and inflammatory endothelial activation, warrant investigation in future studies with more granular mechanistic data.

### Study limitations

We note several limitations of this study that warrant consideration. First, the retrospective observational design introduces the possibility of selection bias and unmeasured confounding, despite our application of PSM and multivariable Cox regression to minimise baseline differences and adjust for residual imbalances. In addition, the use of complete-case analysis may have introduced selection bias, as 417 otherwise eligible patients were excluded due to missing data. We did not formally assess variable-specific patterns or mechanisms of missingness or compare excluded patients with those included, limiting our ability to evaluate the extent of potential selection bias. Second, we assessed AR severity by transthoracic echocardiography during the index hospitalisation but did not reassess it during follow-up. In addition, as we did not perform central image rereading or formal inter- and intra-observer reproducibility assessment, some degree of inter-observer variability and misclassification, particularly between trivial and mild AR, cannot be excluded. Given that AR severity may progress over time, particularly in the context of ongoing hemodynamic stress, aortic root dilation, or degenerative valve disease, the classification of patients as having ‘mild AR’ throughout the entire follow-up period may not accurately reflect their true valvular status at the time of endpoint occurrence. Future prospective studies should incorporate serial echocardiographic reassessments to capture the dynamic nature of VHD. Third, as we did not perform competing risk analyses, we could not account for the competing effect of death for nonfatal outcomes such as HF rehospitalisation and unplanned coronary revascularisation, which may have influenced the estimated event risks. Future studies incorporating competing risk methods are warranted to confirm these associations. Fourth, despite adjustment for a comprehensive set of covariates, data on natriuretic peptide levels (B-type natriuretic peptide/N-terminal pro-B-type natriuretic peptide), coronary microvascular resistance, and serial inflammatory biomarkers during follow-up were not available. The inclusion of such parameters in future studies would substantially enhance mechanistic interpretation. Fifth, the mediation analysis should be considered as exploratory because mild AR and LAD were assessed concurrently, precluding causal inference. The modest indirect associations through LAD (6.8% for MACEs and 10.0% for cardiac death) should be regarded as model-based statistical estimates, rather than evidence of a causal pathway. Prospective studies with serial echocardiographic assessments are required before clinical implications can be established. Sixth, we conducted this study across six tertiary cardiovascular centres in China; therefore, the generalisability of these findings to populations of different ethnicities, healthcare systems, and baseline risk profiles requires external validation in independent cohorts from other geographic regions. Seventh, the associations between mild AR and inflammatory markers do not establish directionality, so reverse causation and residual confounding by underlying coronary or ischaemic burden cannot be excluded. Therefore, the proposed relationship between AR-related hemodynamic stress and inflammation remains speculative and should be considered hypothesis-generating. Eight, the use of a valve-disease-free comparator may introduce residual confounding, as mild AR may partly reflect broader age-related or degenerative cardiovascular changes. This means that we could not establish that the observed prognostic association is specific to mild AR. Future studies comparing mild AR with other mild valvular lesions are warranted. Ninth, residual confounding cannot be completely excluded because cardiac structural and functional parameters were not included in the propensity-score model. Although additional adjustment for LVEF, LVEDD, and LAD yielded consistent results, these variables may reflect baseline disease severity or cardiac remodelling. Finally, the large number of secondary, mediation, biomarker, and subgroup analyses increase the risk of type I error, especially as we did not perform formal multiplicity adjustment. Therefore, findings with *P*-values close to 0.05 should be regarded as exploratory and require confirmation in independent cohorts.

## CONCLUSIONS

In this multicentre propensity score-matched cohort study, mild AR was independently associated with higher risks of MACEs and each of its individual components, including cardiac death, heart failure rehospitalisation, and unplanned coronary revascularisation, after multivariable adjustment in patients with NSTE-ACS. Beyond its prognostic impact, it was also associated with modest differences in systemic inflammatory indices and remnant cholesterol levels, with exploratory analyses suggesting a modest statistical indirect association through LAD. However, the exploratory analyses could not establish a causal mediating role of LAD in the associations between mild AR and MACEs or cardiac death. These findings challenge the traditional perception of mild AR as a hemodynamically inconsequential incidental finding and support its systematic recognition during routine echocardiographic assessment in NSTE-ACS.

## Additional material


Online Supplementary Document

## Data Availability

**Data availability:** De-identified individual participant data underlying the results reported in this article may be made available from the corresponding author upon reasonable request, subject to applicable institutional and ethical requirements.

## References

[1] Berg ES Tegn NK Abdelnoor M Røysland K Ryalen PC Aaberge L Long-Term Outcomes of Invasive vs Conservative Strategies for Older Patients With Non-ST-Segment Elevation Acute Coronary Syndromes. J Am Coll Cardiol. 2023;82:2021–30. 10.1016/j.jacc.2023.09.809 37968019

[2] Cao D Vranckx P Valgimigli M Sartori S Angiolillo DJ Bangalore S One- versus three-month dual antiplatelet therapy in high bleeding risk patients undergoing percutaneous coronary intervention for non-ST-segment elevation acute coronary syndromes. EuroIntervention. 2024;20:e630–42. 10.4244/EIJ-D-23-00658 38776146 PMC11100501

[3] Rao SV O’Donoghue ML Ruel M Rab T Tamis-Holland JE Alexander JH 2025 ACC/AHA/ACEP/NAEMSP/SCAI Guideline for the Management of Patients With Acute Coronary Syndromes: A Report of the American College of Cardiology/American Heart Association Joint Committee on Clinical Practice Guidelines. J Am Coll Cardiol. 2025;85:2135–237. 10.1016/j.jacc.2024.11.009 40013746

[4] Wu J Yang H Li H Meng W Huo Y Zhang Y Distribution of diseases among emergency Chest Pain Center patients from 2018 to 2022: a retrospective analysis from the Chinese Cardiovascular Association Database. Cardiol Plus. 2025;10:261–8. 10.1097/CP9.0000000000000139

[5] Stamatelopoulos K Mueller-Hennessen M Georgiopoulos G Lopez-Ayala P Sachse M Vlachogiannis NI Cathepsin S Levels and Survival Among Patients With Non-ST-Segment Elevation Acute Coronary Syndromes. J Am Coll Cardiol. 2022;80:998–1010. 10.1016/j.jacc.2022.05.055 36049808

[6] Fox KA Dabbous OH Goldberg RJ Pieper KS Eagle KA Van de Werf F Prediction of risk of death and myocardial infarction in the six months after presentation with acute coronary syndrome: prospective multinational observational study (GRACE). BMJ. 2006;333:1091. 10.1136/bmj.38985.646481.55 17032691 PMC1661748

[7] Duan S Wang J Yu F Song L Liu C Sun J Enrichment of the Postdischarge GRACE Score With Deceleration Capacity Enhances the Prediction Accuracy of the Long-Term Prognosis After Acute Coronary Syndrome. Front Cardiovasc Med. 2022;9:888753. 10.3389/fcvm.2022.888753 35571153 PMC9091655

[8] Nkomo VT Gardin JM Skelton TN Gottdiener JS Scott CG Enriquez-Sarano M Burden of valvular heart diseases: a population-based study. Lancet. 2006;368:1005–11. 10.1016/S0140-6736(06)69208-8 16980116

[9] Coffey S Cox B Williams MJ The prevalence, incidence, progression, and risks of aortic valve sclerosis: a systematic review and meta-analysis. J Am Coll Cardiol. 2014;63 25 Pt A:2852–61. 10.1016/j.jacc.2014.04.018 24814496

[10] d’Arcy JL Coffey S Loudon MA Kennedy A Pearson-Stuttard J Birks J Large-scale community echocardiographic screening reveals a major burden of undiagnosed valvular heart disease in older people: the OxVALVE Population Cohort Study. Eur Heart J. 2016;37:3515–22. 10.1093/eurheartj/ehw229 27354049 PMC5216199

[11] Marigliano AN Ortiz JT Casas J Evangelista A Aortic Regurgitation: From Valvular to Myocardial Dysfunction. J Clin Med. 2024;13:2929. 10.3390/jcm13102929 38792470 PMC11122337

[12] Joyce JJ Joyce TR Ferns SJ Trivial aortic valve regurgitation in children and adolescents with structurally normal hearts: physiologic or pathologic? Cardiol Young. 2024;34:131–6. 10.1017/S1047951123001270 37254631

[13] Vahanian A Beyersdorf F Praz F Milojevic M Baldus S Bauersachs J 2021 ESC/EACTS Guidelines for the management of valvular heart disease. Eur Heart J. 2022;43:561–632. 10.1093/eurheartj/ehab395 34453165

[14] Généreux P Pibarot P Redfors B Mack MJ Makkar RR Jaber WA Staging classification of aortic stenosis based on the extent of cardiac damage. Eur Heart J. 2017;38:3351–8. 10.1093/eurheartj/ehx381 29020232 PMC5837727

[15] Capoulade R Clavel MA Dumesnil JG Chan KL Teo KK Tam JW Impact of metabolic syndrome on progression of aortic stenosis: influence of age and statin therapy. J Am Coll Cardiol. 2012;60:216–23. 10.1016/j.jacc.2012.03.052 22789885

[16] Jones BM Tuzcu EM Krishnaswamy A Popovic Z Mick S Roselli EE Prognostic significance of mild aortic regurgitation in predicting mortality after transcatheter aortic valve replacement. J Thorac Cardiovasc Surg. 2016;152:783–90. 10.1016/j.jtcvs.2016.05.023 27321435

[17] Pibarot P Hahn RT Weissman NJ Monaghan MJ Assessment of paravalvular regurgitation following TAVR: a proposal of unifying grading scheme. JACC Cardiovasc Imaging. 2015;8:340–60. 10.1016/j.jcmg.2015.01.008 25772838

[18] Zoghbi WA Adams D Bonow RO Enriquez-Sarano M Foster E Grayburn PA Recommendations for Noninvasive Evaluation of Native Valvular Regurgitation: A Report from the American Society of Echocardiography Developed in Collaboration with the Society for Cardiovascular Magnetic Resonance. J Am Soc Echocardiogr. 2017;30:303–71. 10.1016/j.echo.2017.01.007 28314623

[19] Pawar S Allen C Mori M Markel J Patel K Thornton G Contemporary Diagnosis and Treatment of Aortic Regurgitation: A State-of-the-Art Review. J Am Coll Cardiol. 2026;87:385–413. 10.1016/j.jacc.2025.10.026 41194752

[20] Libby P Ridker PM Maseri A Inflammation and atherosclerosis. Circulation. 2002;105:1135–43. 10.1161/hc0902.104353 11877368

[21] Ridker PM Bhatt DL Pradhan AD Glynn RJ MacFadyen JG Nissen SE Inflammation and cholesterol as predictors of cardiovascular events among patients receiving statin therapy: a collaborative analysis of three randomised trials. Lancet. 2023;401:1293–301. 10.1016/S0140-6736(23)00215-5 36893777

[22] Ge J New insights into atherosclerotic cardiovascular disease through the lens of metabolism: expanding our approach. Cardiol Plus. 2025;10:1–4. 10.1097/CP9.0000000000000112

[23] Wang J Wang Y Duan S Xu L Xu Y Yin W Multimodal Data-Driven Prognostic Model for Predicting Long-Term Prognosis in Patients With Ischemic Cardiomyopathy and Heart Failure With Preserved Ejection Fraction After Coronary Artery Bypass Grafting: A Multicenter Cohort Study. J Am Heart Assoc. 2024;13:e036970. 10.1161/JAHA.124.036970 39604036 PMC11681600

[24] Tang L Wu M Xu Y Zhu T Fang C Ma K Multimodal data-driven prognostic model for predicting new-onset ST-elevation myocardial infarction following emergency percutaneous coronary intervention. Inflamm Res. 2023;72:1799–809. 10.1007/s00011-023-01781-5 37644338

[25] Varbo A Benn M Tybjærg-Hansen A Jørgensen AB Frikke-Schmidt R Nordestgaard BG Remnant cholesterol as a causal risk factor for ischemic heart disease. J Am Coll Cardiol. 2013;61:427–36. 10.1016/j.jacc.2012.08.1026 23265341

[26] Nordestgaard BG Triglyceride-Rich Lipoproteins and Atherosclerotic Cardiovascular Disease: New Insights From Epidemiology, Genetics, and Biology. Circ Res. 2016;118:547–63. 10.1161/CIRCRESAHA.115.306249 26892957

[27] Libby P Loscalzo J Ridker PM Farkouh ME Hsue PY Fuster V Inflammation, Immunity, and Infection in Atherothrombosis: JACC Review Topic of the Week. J Am Coll Cardiol. 2018;72:2071–81. 10.1016/j.jacc.2018.08.1043 30336831 PMC6196735

[28] Gimbrone MA Jr García-Cardeña G Endothelial Cell Dysfunction and the Pathobiology of Atherosclerosis. Circ Res. 2016;118:620–36. 10.1161/CIRCRESAHA.115.306301 26892962 PMC4762052

[29] Bassand JP Hamm CW Ardissino D Boersma E Budaj A Fernandez-Aviles F Guidelines for the diagnosis and treatment of non-ST-segment elevation acute coronary syndromes. Eur Heart J. 2007;28:1598–660. 10.1093/eurheartj/ehm161 17569677

[30] Praz F Borger MA Lanz J Marin-Cuartas M Abreu A Adamo M 2025 ESC/EACTS Guidelines for the management of valvular heart disease. Eur Heart J. 2025;46:4635–736. 10.1093/eurheartj/ehaf194 40878295

[31] Tsampasian V Militaru C Parasuraman SK Loudon BL Lowery C Rudd A Prevalence of asymptomatic valvular heart disease in the elderly population: a community-based echocardiographic study. Eur Heart J Cardiovasc Imaging. 2024;25:1051–8. 10.1093/ehjci/jeae127 38925103 PMC11288748

[32] Abdurashidova T Monney P Tzimas G Soborun N Regamey J Daux A Non-severe aortic regurgitation increases short-term mortality in acute heart failure with preserved ejection fraction. ESC Heart Fail. 2020;7:3901–9. 10.1002/ehf2.12983 33026164 PMC7754983

[33] De Colle C Paolisso P Gallinoro E Bertolone DT Mileva N Fabbricatore D Association of Mild-to-Moderate Aortic Regurgitation With Outcomes in Heart Failure With Preserved Ejection Fraction. Mayo Clin Proc. 2023;98:1469–81. 10.1016/j.mayocp.2023.06.017 37793725

[34] Anand V Michelena HI Pellikka PA Noninvasive Imaging for Native Aortic Valve Regurgitation. J Am Soc Echocardiogr. 2024;37:1167–81. 10.1016/j.echo.2024.08.009 39218370

[35] Shiono Y Matsuo H Fujita H Tanaka N Ogasawara Y Kawamura I Diagnostic Accuracy of Diastolic Fractional Flow Reserve for Functional Evaluation of Coronary Stenosis: DIASTOLE Study. JACC Asia. 2021;1:230–41. 10.1016/j.jacasi.2021.07.008 36338166 PMC9627917

[36] Sengupta A Lee JM Gauvreau K Colan SD Del Nido PJ Mayer JE Jr Natural history of aortic root dilatation and pathologic aortic regurgitation in tetralogy of Fallot and its morphological variants. J Thorac Cardiovasc Surg. 2023;166:1718–1728.e4. 10.1016/j.jtcvs.2023.04.014 37164053

[37] Wenzel JP Petersen E Nikorowitsch J Müller J Kölbel T Reichenspurner H Aortic root dimensions as a correlate for aortic regurgitation’s severity. Int J Cardiovasc Imaging. 2021;37:3439–49. 10.1007/s10554-021-02337-6 34232460 PMC8604845

[38] Covaciu A Benedek T Bobescu E Rus H Benza V Marceanu LG Polyunsaturated Fatty Acids Improved Long Term Prognosis by Reducing Oxidative Stress, Inflammation, and Endothelial Dysfunction in Acute Coronary Syndromes. Mar Drugs. 2025;23:154. 10.3390/md23040154 40278275 PMC12029113

[39] Spindler JS Henning RJ The critical role of inflammation in atherosclerotic coronary artery heart disease. Curr Probl Cardiol. 2026;51:103261. 10.1016/j.cpcardiol.2026.103261 41539582

[40] Wang Y Wang Y Luan Y Hao M Yin W Wu B Multimodal Data-Driven Explainable Prognostic Model for Major Adverse Cardiovascular Events Prediction in Patients With Unstable Angina and Heart Failure With Preserved Ejection Fraction: Multicenter, Cross-Regional Cohort Study. J Med Internet Res. 2025;27:v27i12e78402. 10.2196/78402 41397298 PMC12705130

[41] Zhou B Tan W Duan S Wang Y Bian F Zhao P Inflammation Biomarker-Driven Vertical Visualization Model for Predicting Long-Term Prognosis in Unstable Angina Pectoris Patients with Angiographically Intermediate Coronary Lesions. J Inflamm Res. 2024;17:10571–84. 10.2147/JIR.S497546 39659751 PMC11629667

[42] Bao Q Liu T Song H Bao W Fan W Prognostic Role of Inflammatory Hematologic Indices in Predicting Acute Coronary Syndrome in Elderly Patients with Chronic Coronary Syndrome. J Inflamm Res. 2025;18:9637–53. 10.2147/JIR.S528161 40727457 PMC12301150

[43] Malina M Banasiak W Doroszko A Aortic Valve Defect as an Independent Risk Factor for Endothelial Dysfunction. Cells. 2025;14:885. 10.3390/cells14120885 40558512 PMC12190538

[44] Hmadeh S Trimaille A Matsushita K Marchandot B Carmona A Zobairi F Human Aortic Stenotic Valve-Derived Extracellular Vesicles Induce Endothelial Dysfunction and Thrombogenicity Through AT1R/NADPH Oxidases/SGLT2 Pro-Oxidant Pathway. JACC Basic Transl Sci. 2024;9:845–64. 10.1016/j.jacbts.2024.02.012 39170957 PMC11334416

[45] Abd Alamir M Goyfman M Johnson D Liu Y Dabbous F Chaus A The relationship between endothelial function and aortic valve calcification: Multi-Ethnic Study of Atherosclerosis. Atherosclerosis. 2019;280:155–65. 10.1016/j.atherosclerosis.2018.11.029 30529828

[46] Yang PT Li Y Wang JG Zhang LJ Yang SQ Tang L The Association of Remnant Cholesterol with Endothelial Dysfunction and Subclinical Atherosclerosis in a Check-Up Population in China. J Atheroscler Thromb. 2023;30:684–97. 10.5551/jat.63695 36104205 PMC10244076

[47] Li Y Zhai Y Hu S Liu J Zhang W Yue J Remnant cholesterol, lipid ratios, and the severity of coronary artery lesions: a retrospective cohort study in patients with coronary heart disease. Front Cardiovasc Med. 2025;12:1516326. 10.3389/fcvm.2025.1516326 40129766 PMC11930836

[48] Bernelot Moens SJ Verweij SL Schnitzler JG Stiekema LCA Bos M Langsted A Remnant Cholesterol Elicits Arterial Wall Inflammation and a Multilevel Cellular Immune Response in Humans. Arterioscler Thromb Vasc Biol. 2017;37:969–75. 10.1161/ATVBAHA.116.308834 28336558

[49] Wadström BN Pedersen KM Wulff AB Nordestgaard BG Elevated remnant cholesterol, plasma triglycerides, and cardiovascular and non-cardiovascular mortality. Eur Heart J. 2023;44:1432–45. 10.1093/eurheartj/ehac822 36631967

[50] Johansen MØ Vedel-Krogh S Nielsen SF Afzal S Davey Smith G Nordestgaard BG Association of remnant cholesterol with unhealthy lifestyle and risk of coronary heart disease: a population-based cohort study. Lancet Reg Health Eur. 2025;51:101223. 10.1016/j.lanepe.2025.101223 40213499 PMC11984604

[51] Varbo A Nordestgaard BG Remnant Cholesterol and Triglyceride-Rich Lipoproteins in Atherosclerosis Progression and Cardiovascular Disease. Arterioscler Thromb Vasc Biol. 2016;36:2133–5. 10.1161/ATVBAHA.116.308305 27784698

[52] Tardif JC Kouz S Waters DD Bertrand OF Diaz R Maggioni AP Efficacy and Safety of Low-Dose Colchicine after Myocardial Infarction. N Engl J Med. 2019;381:2497–505. 10.1056/NEJMoa1912388 31733140

[53] Rosenberg MA Manning WJ Diastolic dysfunction and risk of atrial fibrillation: a mechanistic appraisal. Circulation. 2012;126:2353–62. 10.1161/CIRCULATIONAHA.112.113233 23129702

[54] Tsang TS Barnes ME Gersh BJ Bailey KR Seward JB Left atrial volume as a morphophysiologic expression of left ventricular diastolic dysfunction and relation to cardiovascular risk burden. Am J Cardiol. 2002;90:1284–9. 10.1016/S0002-9149(02)02864-3 12480035

