# Supplementary material for: Mild aortic regurgitation as an independent predictor of major adverse cardiovascular events in non-ST-elevation acute coronary syndrome: insights from a multicentre propensity score-matched cohort study
Source: J Glob Health. 2026 Nov 13;16:04404. doi: 10.7189/jogh.16.04404 (PMC13761199; doi:10.7189/jogh.16.04404)
Supplement: Supplementary file 1 [file jogh-16-04404-s001.pdf]

Supplement to: Zhao P, Fang W, Liu H, Zhou B, Zhu T, Zhu J, Tang L, Wu B, Wu S, Wang J, Liu J. Mild aortic regurgitation as an independent predictor of major adverse cardiovascular events in non-ST-elevation acute coronary syndrome: insights from a multicentre propensity score-matched cohort study. J Glob Health. 2026;16:04404.

Table S1. Univariate Cox regression analysis of clinical outcomes in the propensity score–matched NSTE-ACS cohort

Variables	MACEs HR (95%Cl)	P value	Cardiac death HR (95%Cl)	P value	Revascularization HR (95%Cl)	P value	Heart Failure Rehospitalization HR (95%Cl)	P value
Male sex, n (%)	0.982 [0.875;1.126]	0.799	0.972 [0.750;1.260]	0.831	1.018 [0.754;1.374]	0.910	0.972 [0.803;1.177]	0.771
Age, years	1.000 [0.995-1.006]	0.885	1.002 [0.991;1.013]	0.781	1.004 [0.991;1.017]	0.520	0.998 [0.999;1.006]	0.602
Hypertension, n (%)	0.927 [0.807-1.065]	0.285	0.834 [0.644;1.081]	0.171	0.894 [0.661;1.209]	0.467	1.006 [0.826;1.224]	0.954
Diabetes mellitus, n (%)	1.328 [1.156-1.525]	<0.001	1.376 [1.061;1.785]	0.016	1.412 [1.046;1.904]	0.024	1.236 [1.017;1.502]	0.033
Smoking history, n (%)	1.173 [1.021-1.347]	0.024	1.325 [1.024;1.716]	0.033	1.443 [1.074;1.940]	0.015	0.990 [0.812;1.208]	0.924
Alcohol consumption history, n (%)	0.973 [0.834-1.136]	0.731	0.977 [0.728;1.310]	0.875	1.021 [0.731;1.426]	0.904	0.959 [0.771;1.191]	0.704
Family history of CAD, n (%)	0.996 [0.707-1.404]	0.983	1.061 [0.563;1.998]	0.854	0.830 [0.368;1.872]	0.653	1.039 [0.648;1.665]	0.873
Previous myocardial infarction, n (%)	0.774 [0.541-1.107]	0.161	1.018 [0.556;1.864]	0.954	0.772 [0.320;1.628]	0.432	0.684 [0.401;1.164]	0.161
Unstable angina, n (%)	0.956 [0.771-1.184]	0.677	0.871 [0.571;1.327]	0.519	0.757 [0.453;1.265]	0.288	1.092 [0.822;1.452]	0.543

BMI,Kg/m^2	1.027 [1.006-1.047]	0.010	1.064 [1.008;1.085]	0.017	1.052 [1.008;1.097]	0.020	1.003 [0.975;1.032]	0.836
Use of Aspirin, n (%)	0.887 [0.754-1.042]	0.145	0.896 [0.659;1.218]	0.482	1.029 [0.712;1.487]	0.880	0.839 [0.672;1.048]	0.122
Use of Clopidogrel, n (%)	1.046 [0.879-1.246]	0.613	0.879 [0.619;1.247]	0.469	0.999 [0.679;1.471]	0.997	1.155 [0.912;1.463]	0.231
Use of Ticagrelor, n (%)	1.410 [0.846-2.350]	0.187	1.597 [0.659;3.873]	0.300	1.300 [0.415;4.070]	0.652	1.262 [0.598;2.663]	0.541
Use of Statins, n (%)	0.934 [0.799-1.091]	0.390	1.198 [0.873-1.643]	0.262	0.902 [0.644;1.263]	0.547	0.844 [0.683;1.044]	0.117
Use of Beta blockers, n (%)	0.931 [0.815-1.064]	0.291	0.925 [0.718;1.191]	0.545	1.002 [0.749;1.340]	0.992	0.908 [0.754;1.095]	0.313
Use of ACEI, n (%)	0.943 [0.758-1.173]	0.598	1.033 [0.692;1.541]	0.876	1.131 [0.724;1.766]	0.588	0.829 [0.601;1.143]	0.253
Use of ARB, n (%)	0.913 [0.773-1.079]	0.286	0.970 [0.711;1.325]	0.850	0.854 [0.588;1.240]	0.406	0.921 [0.730;1.161]	0.485
Use of CCB, n (%)	1.099 [0.947-1.276]	0.215	1.187 [0.899;1.567]	0.228	1.170 [0.848;1.614]	0.339	1.025 [0.830;1.267]	0.818
Use of ARNI, n (%)	1.028 [0.842-1.256]	0.786	0.663 [0.424;1.037]	0.072	1.116 [0.732;1.701]	0.611	1.184 [0.908;1.542]	0.212
SBP, mmHg	1.000 [0.997;1.003]	0.924	0.997 [0.992;1.003]	0.305	0.996 [0.990;1.002]	0.227	1.003 [0.999;1.007]	0.097
DBP, mmHg	1.003 [0.998;1.007]	0.247	1.001 [0.993;1.010]	0.733	1.003 [0.993;1.013]	0.556	1.003 [0.997;1.010]	0.316
White blood count, $\times 10^9$/L	0.988 [0.960;1.017]	0.407	0.979 [0.926;1.035]	0.449	0.980 [0.920;1.045]	0.543	0.997 [0.958;1.037]	0.868
Neutrophil count, $\times 10^9$/L	0.989 [0.958;1.020]	0.467	0.974 [0.916;1.034]	0.388	0.977 [0.912;1.047]	0.514	1.002 [0.960;1.045]	0.940
Lymphocyte count, $\times 10^9$/L	1.051 [0.960;1.150]	0.280	1.068 [0.902;1.264]	0.444	0.998 [0.815;1.223]	0.984	1.061 [0.937;1.203]	0.351
Monocyte count, $\times 10^9$/L	1.172 [0.869;1.581]	0.298	1.066 [0.601;1.890]	0.827	1.460 [0.774;2.755]	0.242	1.100 [0.722;1.675]	0.658
Platelet count, $\times 10^9$/L	1.000 [0.999;1.001]	0.455	0.999 [0.997;1.001]	0.485	1.001 [0.999;1.003]	0.280	1.001 [0.999;1.002]	0.422

NLR	0.987 [0.964;1.011]	0.292	0.968 [0.921;1.018]	0.206	0.994 [0.944;1.045]	0.804	0.994 [0.962;1.027]	0.714
SII	1.000 [1.000;1.000]	0.567	1.000 [1.000;1.000]	0.163	1.000 [1.000;1.000]	0.858	1.000 [1.000;1.000]	0.922
PLR	1.000 [0.999;1.001]	0.591	0.999 [0.997;1.000]	0.109	1.001 [0.999;1.002]	0.318	1.000 [0.999;1.001]	0.799
MLR	1.113 [0.887;1.396]	0.356	0.745 [0.411;1.349]	0.331	1.413 [0.999;1.999]	0.051	1.086 [0.803;1.468]	0.592
Creatinine, μmol/L	1.002 [1.000;1.005]	0.045	1.001 [0.997;1.006]	0.618	1.002 [0.997;1.007]	0.439	1.003 [1.000;1.006]	0.074
Uric acid, μmol/L	1.000 [0.999;1.001]	0.838	1.000 [0.999;1.001]	0.896	1.000 [0.999;1.001]	0.873	1.000 [0.999;1.001]	0.668
Blood glucose, mmol/L	1.002 [0.982;1.023]	0.834	0.995 [0.956;1.036]	0.806	0.955 [0.904;1.010]	0.107	1.020 [0.994;1.048]	0.133
Heart rate, times per minute	1.001 [0.996;1.006]	0.670	1.001 [0.991;1.010]	0.887	1.000 [0.989;1.011]	0.986	1.002 [0.995;1.009]	0.599
Total cholesterol, mmol/L	1.043 [0.977;1.114]	0.207	1.066 [0.942;1.206]	0.313	0.935 [0.805;1.086]	0.378	1.073 [0.979;1.175]	0.131
TG, mmol/L	1.001 [0.959;1.044]	0.982	1.001 [0.926;1.082]	0.979	0.940 [0.838;1.055]	0.292	1.019 [0.965;1.076]	0.505
HDL-C, mmol/L	0.908 [0.728;1.133]	0.394	0.945 [0.625;1.431]	0.791	0.743 [0.452;1.218]	0.239	1.057 [0.959;1.165]	0.266
LDL-C, mmol/L	0.977 [0.910;1.049]	0.525	0.942 [0.805;1.060]	0.258	0.871 [0.742;1.024]	0.095	0.968 [0.713;1.315]	0.837
Fibrinogen, g/L	0.998 [0.967;1.030]	0.902	0.958 [0.886;1.036]	0.287	1.020 [0.961;1.082]	0.512	1.003 [0.962;1.047]	0.874
RC, mmol/L	1.107 [1.026;1.195]	0.009	1.376 [1.201;1.408]	0.008	1.115 [0.945;1.316]	0.196	1.037 [0.927;1.160]	0.524
Left ventricular ejection fraction, %	0.993 [0.985;1.002]	0.129	0.992 [0.977;1.007]	0.280	1.005 [0.987;1.023]	0.594	0.992 [0.980;1.004]	0.179
LVEDD, mm	1.008 [0.995;1.021]	0.217	1.017 [0.993;1.042]	0.169	0.986 [0.959;1.014]	0.332	1.011 [0.993;1.029]	0.229
Left atrial diameter, mm	1.024 [1.010;1.039]	0.001	1.042 [1.015;1.071]	0.002	1.013 [0.983;1.045]	0.396	1.017 [0.997;1.037]	0.100

Percutaneous coronary intervention, n (%)	0.946 [0.809;1.107]	0.489	0.957 [0.711;1.288]	0.773	1.026 [0.733;1.437]	0.881	0.908 [0.727;1.133]	0.392
Mild AR, n (%)	1.472 [1.276;1.698]	<0.001	1.508 [1.154;1.971]	0.003	1.568 [1.153;2.133]	0.005	1.342 [1.096;1.643]	0.004
Number of implanted stents, n (%)	1.210 [1.129;1.298]	<0.001	1.127 [0.981;1.295]	0.092	1.463 [1.287;1.664]	<0.001	1.118 [1.008;1.241]	0.035
The number of coronary arteries with≥50% stenosis, n (%)								
Two-vessel CAD vs One-vessel CAD	1.450 [1.175;1.789]	0.001	2.318 [1.653;3.250]	<0.001	2.209 [1.468;3.323]	<0.001	0.761 [0.524;1.107]	0.153
Three-vessel CAD vs One-vessel CAD	1.486 [1.255;1.759]	<0.001	1.463 [1.050;2.040]	0.025	1.851 [1.294;2.647]	0.001	1.367 [1.081;1.728]	0.009

Abbreviations: BMI, body mass index; SBP, systolic blood pressure; DBP, diastolic blood pressure; CAD, coronary artery disease; MI, myocardial infarction; ACEI, angiotensin-converting enzyme inhibitor; ARB, angiotensin receptor blocker; CCB, calcium channel blocker; ARNI, angiotensin receptor neprilysin inhibitor; TG, triglyceride; HDL-C, high-density lipoprotein cholesterol; LDL-C, low-density lipoprotein cholesterol; RC, remnant cholesterol; NLR, neutrophil-to-lymphocyte ratio; SII, systemic immune-inflammation index; PLR, platelet-to-lymphocyte ratio; MLR, monocyte-to-lymphocyte ratio; LVEDD, left ventricular end-diastolic dimension.

Table S2. Mediation effects of cardiac structural parameters, hemodynamic indices, and metabolic variables on the associations of mild AR with MACEs, cardiac death, HF rehospitalization, and unplanned coronary revascularization

Mediators	Natural mediated effect		Total effect		Proportion of mediating effect
	β (95%CI)	*P* value	β (95%CI)	*P* value	
**MACEs**					
LVEDD,mm	0.001(-0.00,0.003)	0.588	0.059(0.035,0.082)	<0.001	1.7%
LAD,mm	0.004(0.001,0.007)	0.008	0.059(0.035,0.082)	<0.001	6.8%
EF,%	-0.001(-0.009,0.008)	0.872	0.059(0.035,0.082)	<0.001	-1.7%
SBP, mmHg	0.000(0.000,0.001)	0.824	0.059(0.035,0.082)	<0.001	0.0%
DBP, mmHg	0.000(-0.001,0.001)	0.968	0.059(0.035,0.082)	<0.001	0.0%
RC, mmol/L	-0.000(0.000,0.002)	0.348	0.059(0.035,0.082)	<0.001	0.0%
**Cardiac death**					
LVEDD,mm	0.001(-0.001,0.002)	0.340	0.020(0.007,0.033)	0.003	5.0%
LAD,mm	0.002(0.001,0.004)	<0.001	0.020(0.007,0.033)	0.003	10.0%
EF,%	0.001(-0.004,0.005)	0.800	0.020(0.007,0.033)	0.003	5.0%
SBP, mmHg	0.000(0.000,0.001)	0.716	0.020(0.007,0.033)	0.003	0.0%
DBP, mmHg	0.000(0.000,0.000)	0.944	0.020(0.007,0.033)	0.003	0.0%
RC, mmol/L	0.000(0.000,0.001)	0.612	0.020(0.007,0.033)	0.003	0.0%
**HF rehospitalization**					
LVEDD,mm	0.001(0.000,0.003)	0.436	0.023(0.005,0.041)	0.011	4.3%
LAD,mm	0.001(-0.001,0.003)	0.236	0.023(0.005,0.041)	0.011	4.3%
EF,%	0.001(-0.001,0.010)	0.840	0.023(0.005,0.041)	0.011	4.3%

SBP, mmHg	0.000 (-0.001,0.000)	0.660	0.023(0.005,0.041)	0.011	0.0%
DBP, mmHg	0.000(0.000,0.000)	0.992	0.023(0.005,0.041)	0.011	0.0%
RC, mmol/L	0.000(0.000,0.001)	0.392	0.023(0.005,0.041)	0.011	0.0%
**Unplanned coronary revascularization**					
LVEDD,mm	-0.001(-0.002,0.000)	0.157	0.016(0.004,0.027)	0.008	-6.3%
LAD,mm	0.000(0.000,0.002)	0.448	0.016(0.004,0.027)	0.008	0.0%
EF,%	-0.002(-0.005,0.001)	0.136	0.016(0.004,0.027)	0.008	-12.5%
SBP, mmHg	0.000(-0.000,0.001)	0.640	0.016(0.004,0.027)	0.008	0.0%
DBP, mmHg	0.000(0.000,0.000)	0.964	0.016(0.004,0.027)	0.008	0.0%
RC, mmol/L	0.000(0.000,0.001)	0.648	0.016(0.004,0.027)	0.008	0.0%

Abbreviations: LVEDD,left ventricular end-diastolic dimension; LAD, left atrial diameter; EF, left ventricular ejection fraction; SBP, systolic blood pressure; DBP, diastolic blood pressure; RC, remnant cholesterol; HF, heart failure.

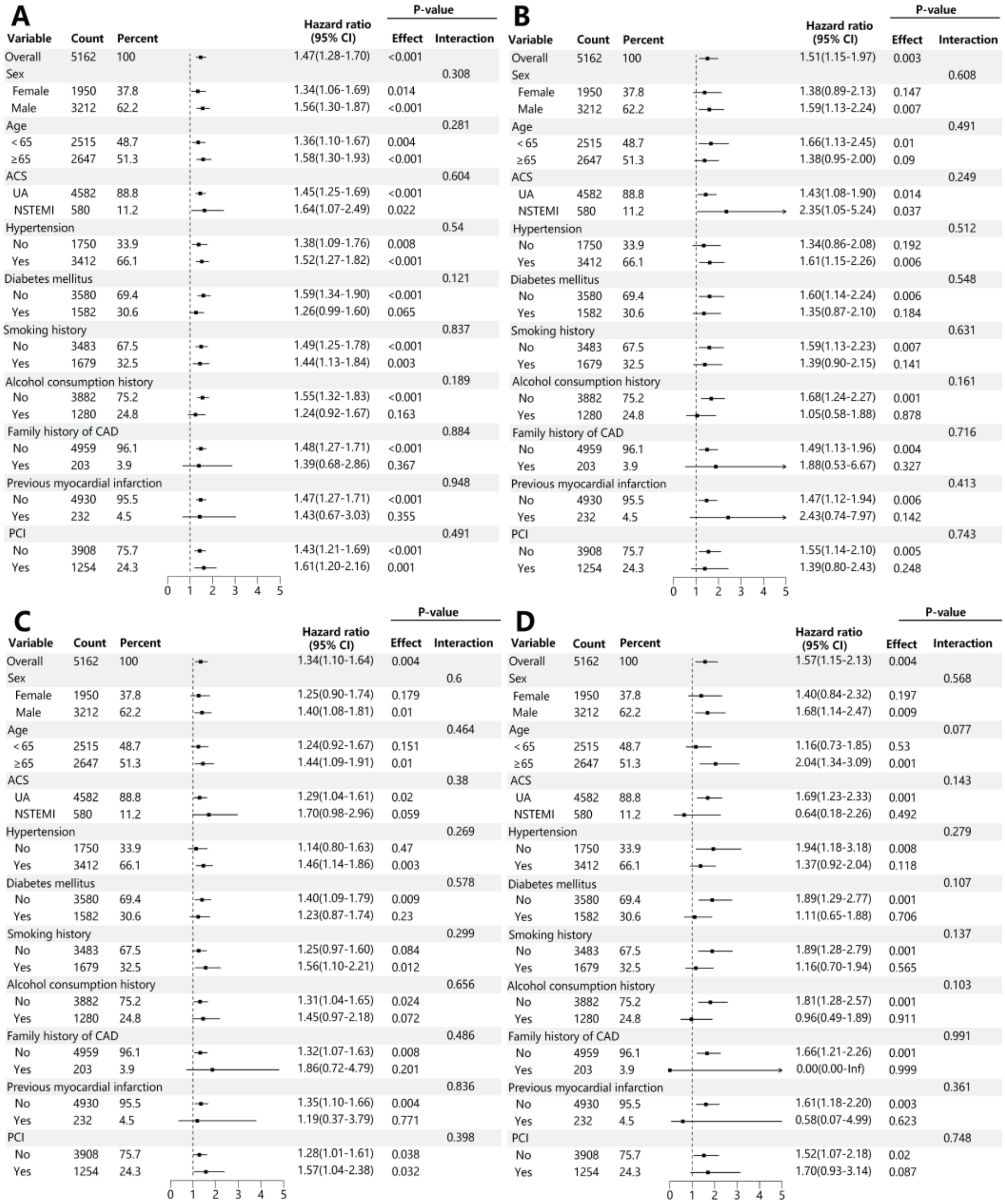

Figure S1. Subgroup analyses of the association between mild AR and MACEs (**Panel A**), cardiac death (**Panel B**), HF rehospitalisation (**Panel C**), and unplanned coronary revascularisation (**Panel D**). ACS – acute coronary syndrome, NSTEMI – non-ST-segment elevation myocardial infarction, PCI – percutaneous coronary intervention, UA – unstable angina.

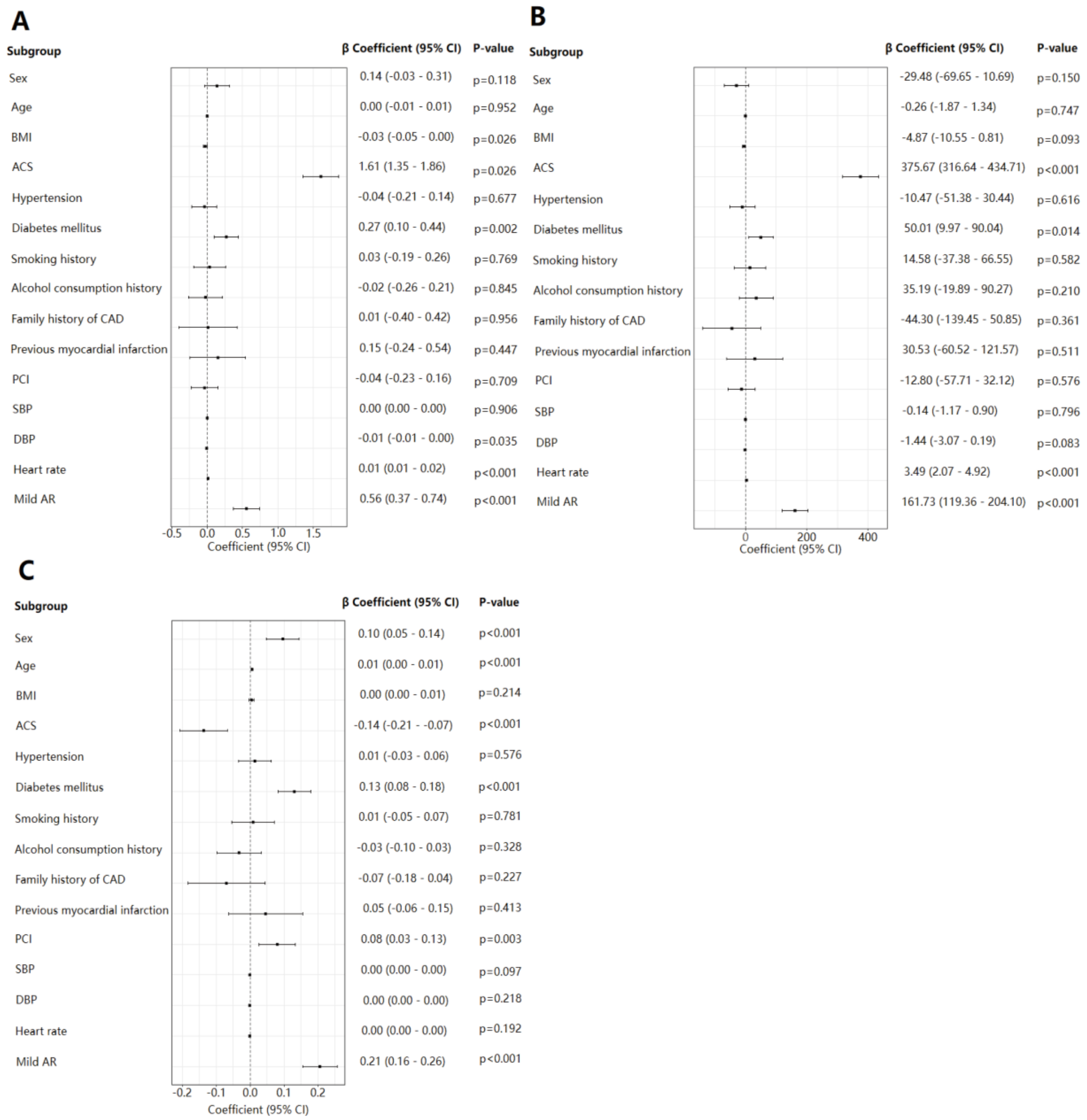

Figure S2. Linear regression analysis of NLR (**Panel A**), SII (**Panel B**), and RC (**Panel C**) in NSTE-ACS patients with mild AR. ACS – acute coronary syndrome, AR – aortic regurgitation, BMI – body mass index, CAD – coronary artery disease, DBP – diastolic blood pressure, PCI – percutaneous coronary intervention, SBP – systolic blood pressure.

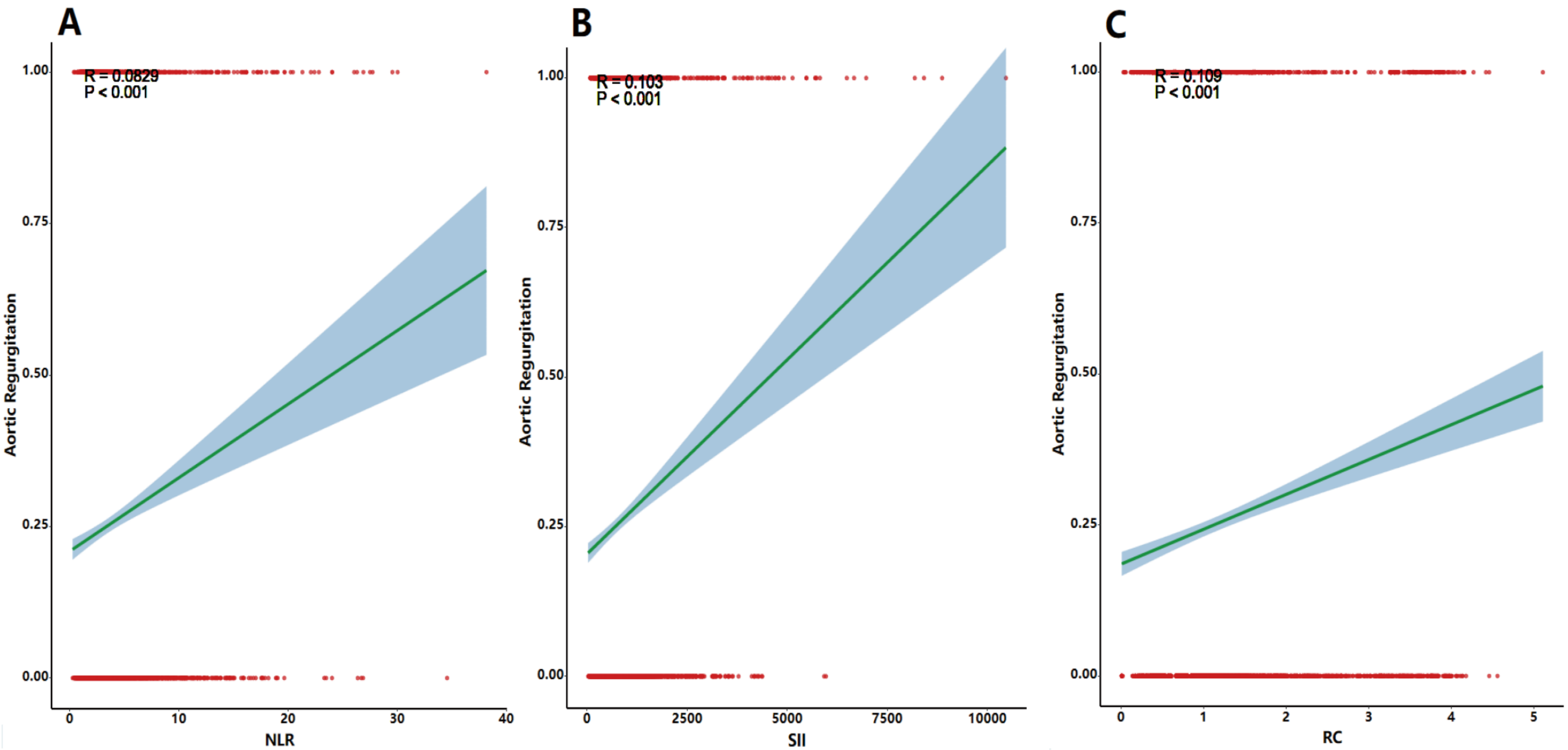

Figure S3. Correlation analysis of mild AR with NLR (**Panel A**), SII (**Panel B**), and RC (**Panel C**). AR – aortic regurgitation, NLR – neutrophil-to-lymphocyte ratio, RC – remnant cholesterol, SII – systemic immune-inflammation index.

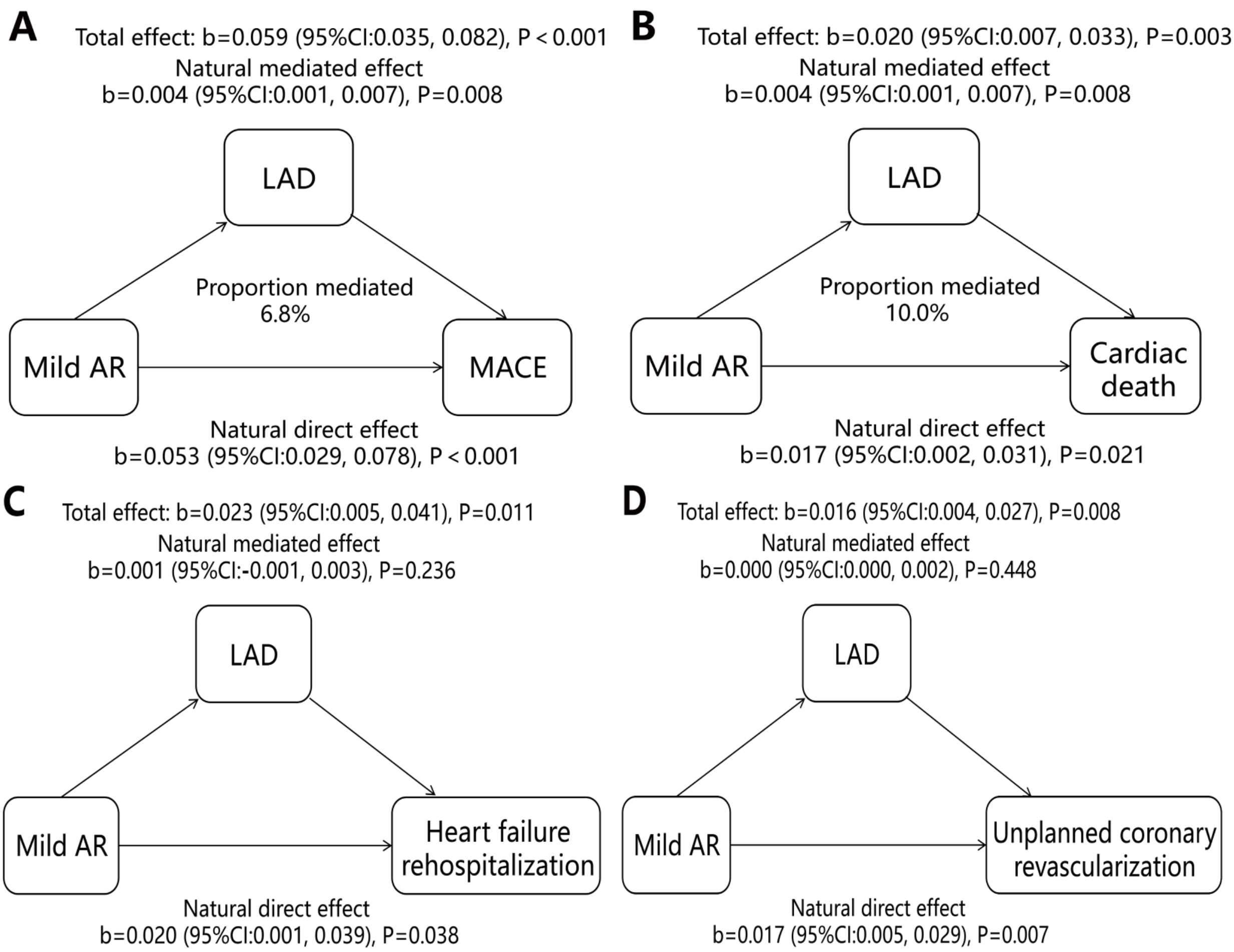

Figure S4. Exploratory mediation analysis of LAD in the associations of mild AR with MACEs (**Panel A**), cardiac death (**Panel B**), HF rehospitalisation (**Panel C**), and unplanned coronary revascularisation (**Panel D**). AR – aortic regurgitation, HF – heart failure, LAD – left atrial diameter, MACEs – major adverse cardiovascular events.
